# Supplementary material for: Akt inhibition enhances the antitumor efficacy of immune checkpoint blockades and radiotherapy in a syngeneic breast cancer model
Source: Mol Ther Oncol. 2025 Nov 11;33(4):201087. doi: 10.1016/j.omton.2025.201087 (PMC12686700; doi:10.1016/j.omton.2025.201087)
Supplement: Document S1. Figures S1–S10 [file mmc1.pdf]

OMTON, Volume *33*

Supplemental information

Akt inhibition enhances the antitumor efficacy of immune checkpoint blockades and radiotherapy in a syngeneic breast cancer model

Nawon Park, Seung Hyuck Jeon, Yoomin Kim, Seongmin Kim, and In Ah Kim

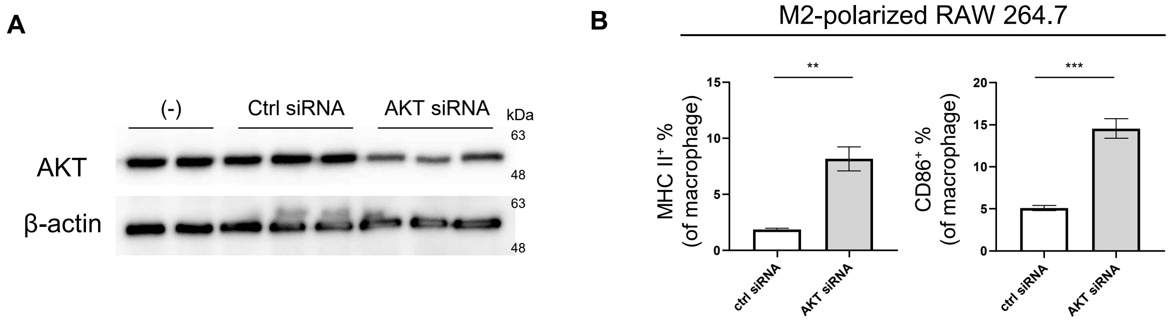

Figure S1. MHCII and CD86 expression following Akt siRNA treatment
RAW 264.7 cells were polarized into M2 macrophages and transfected with Ctrl siRNA or Akt siRNA. (A) Western blot analysis of AKT and β-actin expression. (B) Flow cytometry analysis of MHC II and CD86 expression after siRNA treatment. Positive gating for MHCII and CD86 was defined using M0 macrophages (negative control) with a 5% threshold. Data represent the mean ± S.E.M (n = 4). *P<0.05, **P<0.01, ***P<0.001, ****P<0.0001.

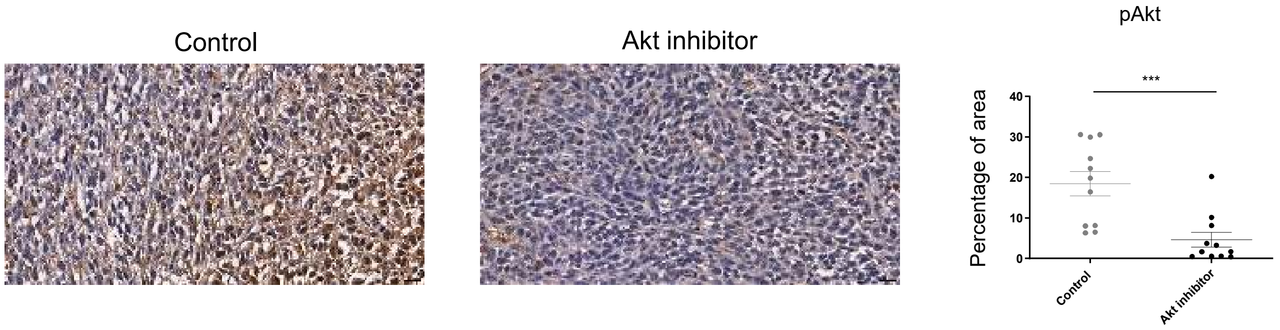

Figure S2. Phospho-Akt (Ser473) expression by IHC in primary tumors.
IHC staining for pAkt in primary tumor sections. Scale bars, 20 μm. Data represent the mean ± S.E.M (n = 11). *P<0.05, **P<0.01, ***P<0.001, ****P<0.0001.

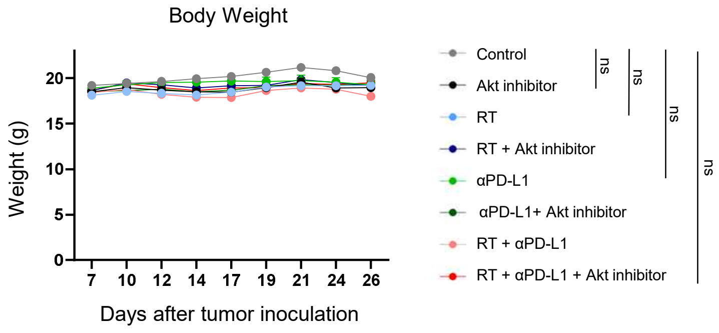

Figure S3. Body weight changes during treatment following RT, αPD-L1 and Akt inhibitor.
Body weight changes during treatment. Data represent the mean ± S.E.M (n = 5). *P<0.05, **P<0.01, ***P<0.001, ****P<0.0001 (two-way ANOVA with multiple comparisons at day 26).

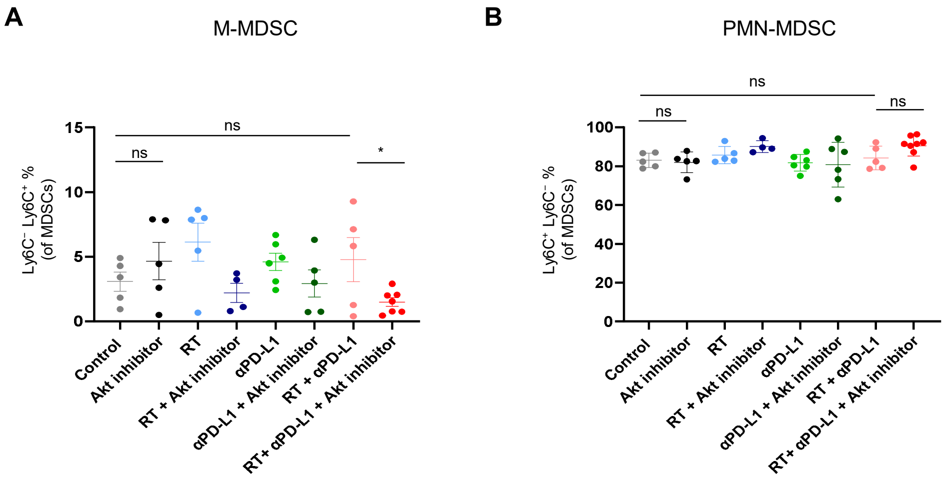

Figure S4. Phenotypic profiling of M-MDSCs and PMN-MDSCs in primary tumors following RT, αPD-L1 and Akt inhibitor.

Frequency of (A) M-MDSC ($CD45^+$, $CD11c^-$, $CD11b^+$, $F4/80^-$, $Ly6G^-$, $Ly6C^+$) and (B) PMN-MDSC ($CD45^+$, $CD11c^-$, $CD11b^+$, $F4/80^-$, $Ly6G^+$, $Ly6C^-$) in primary tumor. All data represent the mean ± S.E.M (n = 4~7). *P<0.05, **P<0.01, ***P<0.001, ****P<0.0001.

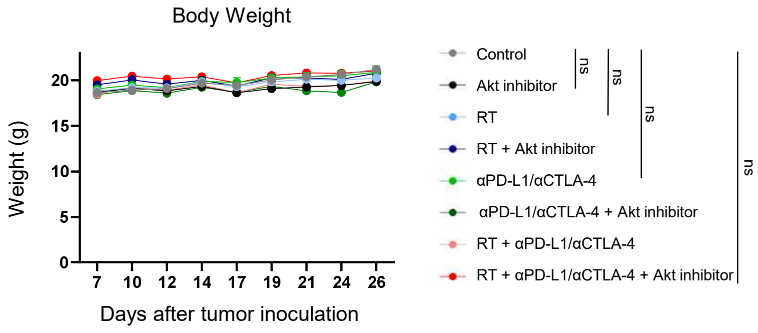

Figure S5. Body weight changes during treatment following RT, αPD-L1/αCTLA-4 and Akt inhibitor. Body weight changes during treatment. All data represent the mean ± S.E.M (n = 5). *P<0.05, **P<0.01, ***P<0.001, ****P<0.0001 (two-way ANOVA with multiple comparisons at day 26).

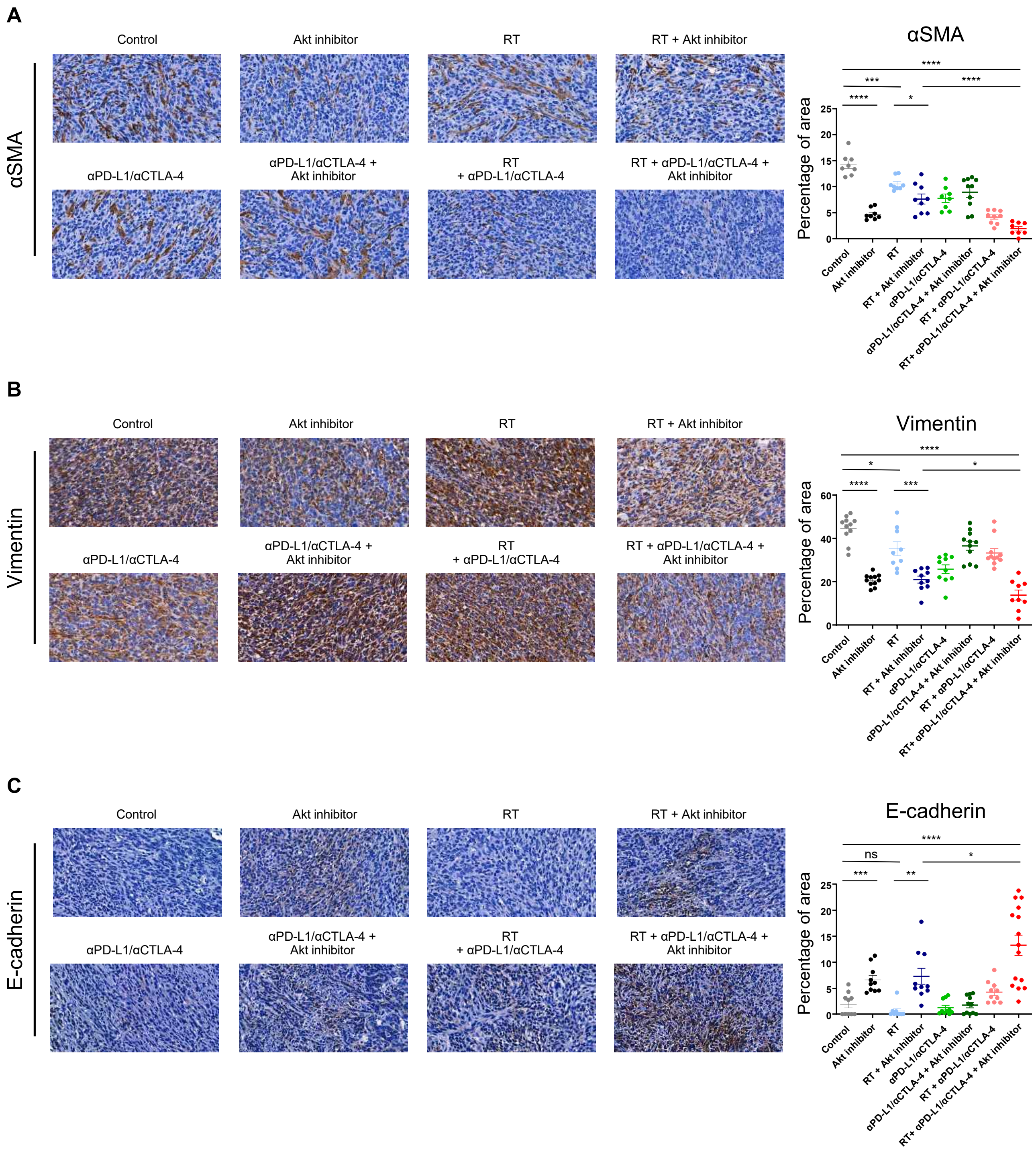

Figure S6. αSMA, vimentin and E-cadherin expression by IHC in primary tumors.
IHC staining of tumor sections for (A) αSMA, (B) vimentin, (C) E-cadherin. Scale bars, 20 μm. All data represent the mean ± S.E.M (n = 8~15). *P<0.05, **P<0.01, ***P<0.001, ****P<0.0001.

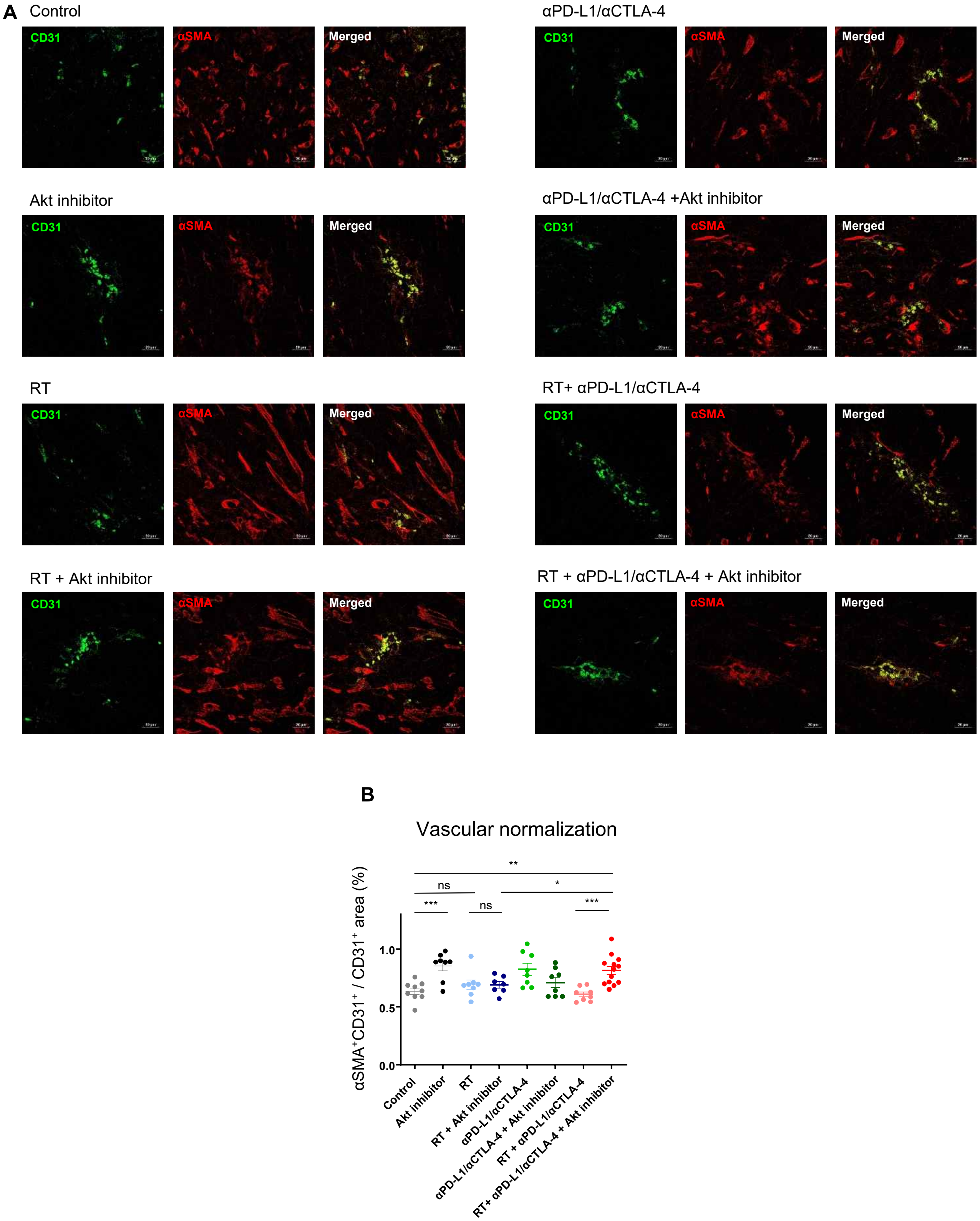

Figure S7. Colocalization of CD31 and αSMA in tumor vasculature.
(A) Immunofluorescence staining images of CD31 (green) and αSMA (red); yellow indicates colocalization. (B) Quantification of $αSMA^{+}CD31^{+}/CD31^{+}$ areas. Scale bars, 20 μm. All data represent the mean ± S.E.M (n = 7~13). *P*<0.05, **P*<0.01, ***P*<0.001, ****P*<0.0001.

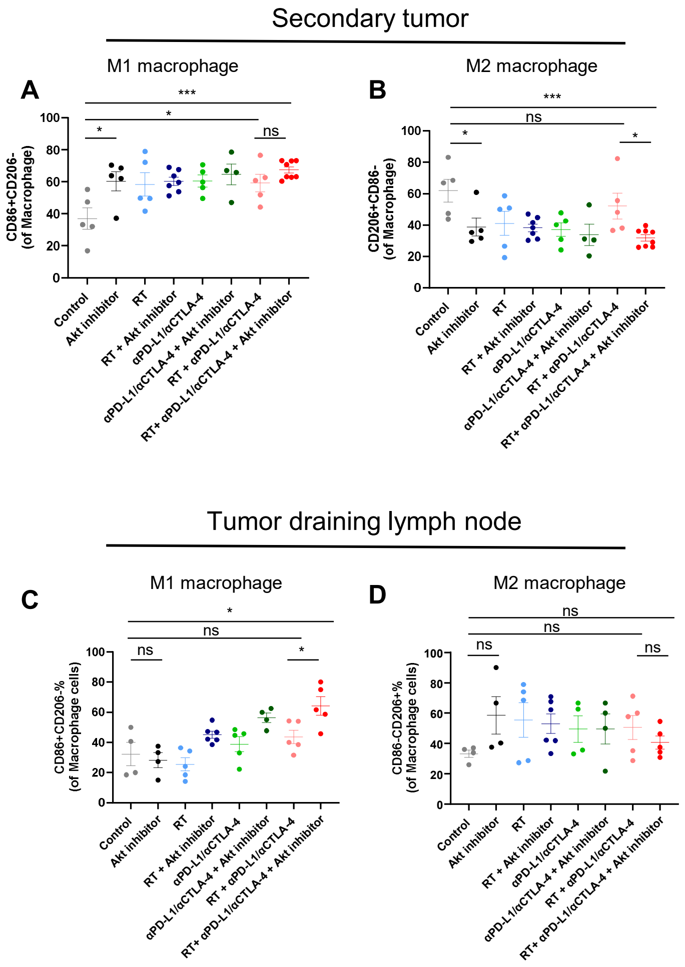

Figure S8. Macrophage profiling in the secondary tumor and tumor draining lymph node following RT, αPD-L1/αCTLA-4 and Akt inhibitor.
Frequency of (A) M1 and (B) M2 macrophages in the secondary tumor. Frequency of (C) M1, (D) M2 macrophages in the tumor-draining lymph node. All data represent the mean ± S.E.M (n = 4~6). *P<0.05, **P<0.01, ***P<0.001, ****P<0.0001.

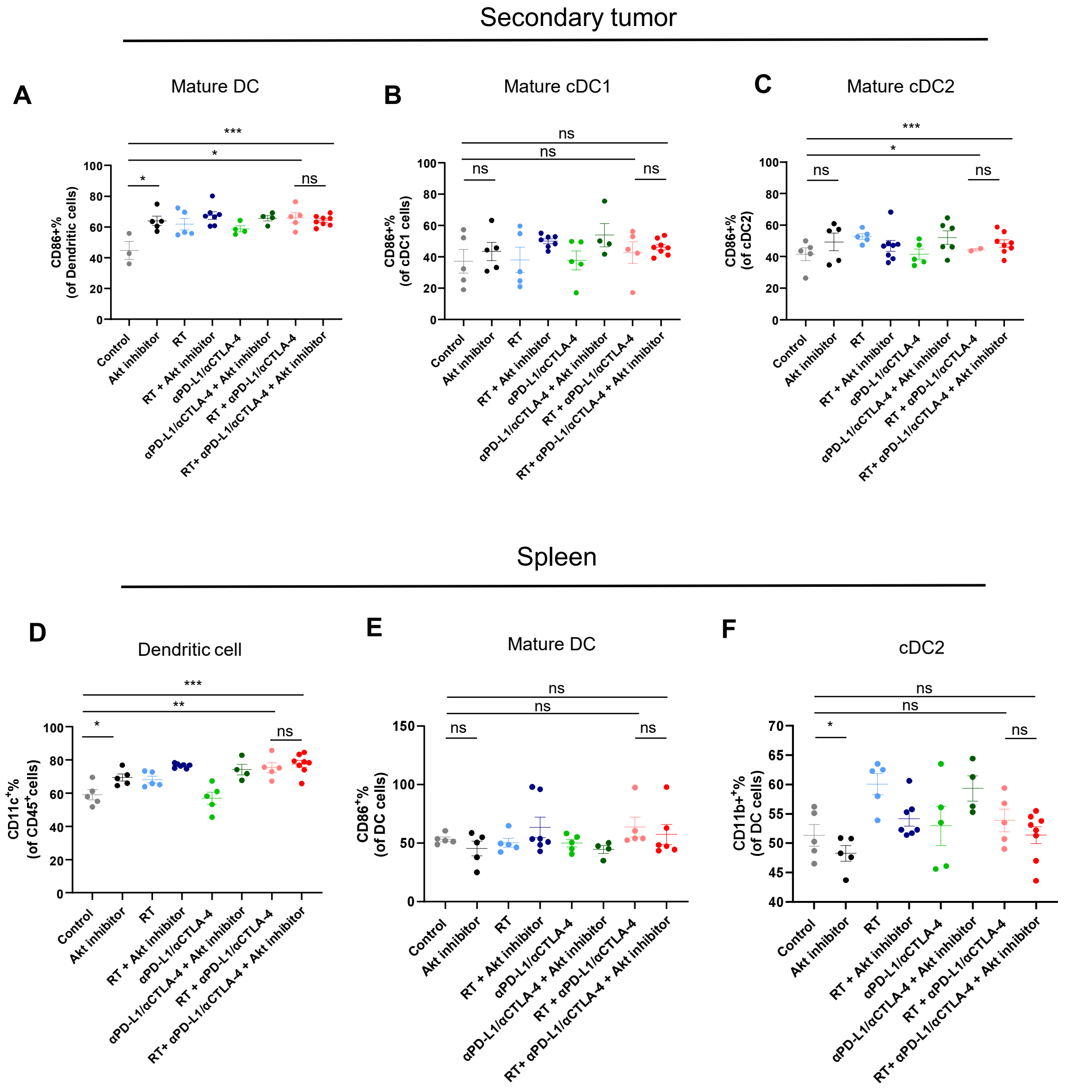

Figure S9. DCs profiling in the secondary tumor and spleen following RT, αPD-L1/αCTLA-4 and Akt inhibitor. Frequency of (A) $CD86^+$ dendritic cells, (B) Frequency of $CD86^+$ cDC1, (C) Frequency of $CD86^+$ cDC2 in the secondary tumor. Frequency of (D) Dendritic cell ($CD45^+$, $CD11c^+$), (E) $CD86^+$ DC, (F) cDC2 in the spleen. All data represent the mean ± S.E.M (n = 4~8). *P<0.05, **P<0.01, ***P<0.001, ****P<0.0001.

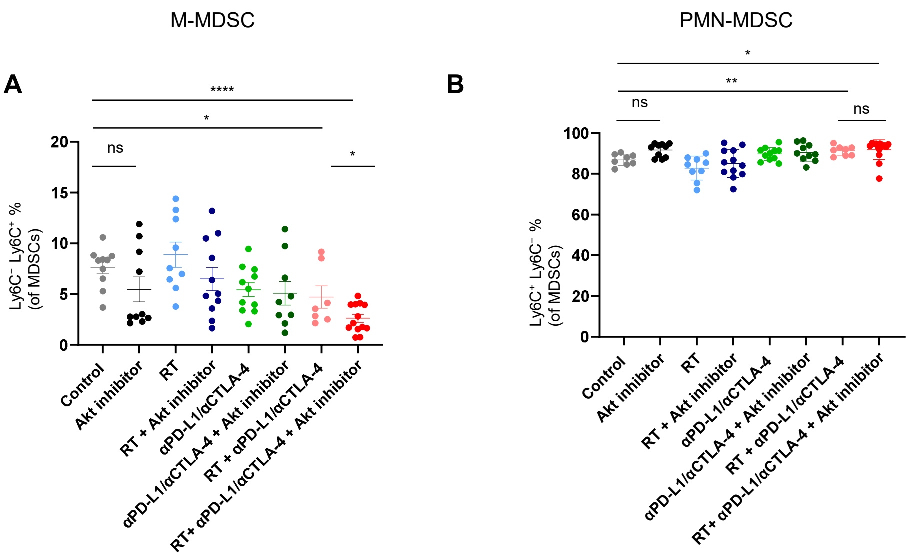

Figure S10. Phenotypic profiling of M-MDSCs and PMN-MDSCs in primary tumors following RT, αPD-L1/αCTLA-4 and Akt inhibitor.

Frequency of (A) M-MDSC and (B) PMN-MDSC in primary tumor. All data represent the mean ± S.E.M (n = 7~13). *P<0.05, **P<0.01, ***P<0.001, ****P<0.0001.
