# Supplementary material for: Akt inhibition enhances the antitumor efficacy of immune checkpoint blockades and radiotherapy in a syngeneic breast cancer model
Source: Mol Ther Oncol. 2025 Nov 11;33(4):201087. doi: 10.1016/j.omton.2025.201087 (PMC12686700; doi:10.1016/j.omton.2025.201087)
Supplement: Document S2. Article plus supplemental information [file mmc2.pdf]

Molecular Therapy
Oncology
Original Article

Akt inhibition enhances the antitumor efficacy of immune checkpoint blockades and radiotherapy in a syngeneic breast cancer model

Nawon Park,[1,2,3,6] Seung Hyuck Jeon,[3,6] Yoomin Kim,[1,2,3] Seongmin Kim,[1,2,3,4] and In Ah Kim[1,2,3,4,5]

[1]Department of Tumor Biology and Cancer Research Institute, Graduate School of Medicine, Seoul National University, Seoul, Republic of Korea; [2]Medical Science Research Institute, Seoul National University Bundang Hospital, Seongnam, Republic of Korea; [3]Department of Radiation Oncology, Seoul National University Bundang Hospital, Seongnam, Republic of Korea; [4]Integrated Major in Innovative Medical Science, Seoul National University, Seoul, Republic of Korea; [5]Department of Radiation Oncology, Seoul National University, Seoul, Republic of Korea

Immune checkpoint blockades (ICBs) have limited efficacy against immunologically cold breast cancers. Although radiotherapy (RT) can potentiate antitumor immunity, many patients fail to benefit, partly due to activation of immunosuppressive cells. Given the ability of PI3K inhibition to regulate immunosuppressive repertoires, we hypothesized that inhibition of Akt, a key effector of PI3K, could enhance the efficacy of RT and ICBs. In this study, we investigated antitumor effects and immune cell phenotypes in 4T1 syngeneic triple-negative breast cancer (TNBC) models treated with RT, ICBs, and an Akt inhibitor. RT + αPD-L1 + Akt inhibitor most reduced tumor size and lung metastases. The Akt inhibitor increased the M1/M2 macrophage ratio and the proportion of CD86$^+$ dendritic cells (DCs). Interestingly, RT + αPD-L1 increased M2 macrophages, while adding the Akt inhibitor partially restored M1/M2 balance. When combined with RT + αPD-L1/αCTLA-4, Akt inhibition further elevated the M1/M2 ratio and CD86$^+$ DC frequency and reduced monocytic myeloid-derived suppressor cells (M-MDSCs). These findings suggest that addition of an Akt inhibitor to RT and ICBs leads to a less immunosuppressive tumor microenvironment by modulating myeloid cells. Taken together, Akt inhibition could be a viable strategy to overcome therapeutic resistance of ICBs and RT in breast cancer.

INTRODUCTION

Immune checkpoint blockades (ICBs) can provide effective treatment for many solid tumors. However, ICBs alone have shown limited efficacy for immunologically cold tumors such as breast cancers.[1,2] Radiotherapy (RT) is regarded as an attractive combination partner of ICB and has shown synergistic effects with ICB by activating anti-tumor immune responses.[3–6] However, both ICB and RT are also known to enhance immunosuppressive cells, including tumor-associated macrophages (TAMs) and myeloid-derived suppressor cells (MDSCs).[7] Therefore, targeting immunosuppressive cells is expected to increase the efficacy of ICB and RT.

Myeloid cells are extremely heterogeneous. They play crucial roles within the tumor microenvironment (TME).[8,9] Especially, TAMs are abundant among myeloid cells infiltrating solid tumors. A considerable portion of TAMs can acquire the phenotype of anti-inflammatory M2 macrophages.[10–12] TAMs are known to promote tumor growth, invasion, and metastasis by secreting anti-inflammatory cytokines and upregulating immunosuppressive molecules.[13,14] Consequently, therapies that can inhibit TAMs have shown ability to enhance anti-tumor effects of chemotherapy, RT, and immunotherapy.[15–17] Other myeloid-lineage immune cells, including dendritic cells (DCs) and MDSCs, can also exert significant impacts on anti-tumor immune responses. Novel strategies to modulate these cells are under active investigation.[18,19]

The PI3K-AKT pathway plays a central role in promoting tumor cell survival, metastasis, and proliferation.[20,21] This pathway is known to contribute to radiation resistance of various tumor cells. Inhibition of key PI3K/AKT components has been shown to be able to enhance the radiosensitivity of tumor cells.[22] These findings suggest the therapeutic potential of PI3K/AKT inhibition when combined with RT.

Growing evidence suggests that PI3K inhibitors can also influence the tumor immune microenvironment, expanding their therapeutic potential beyond direct tumor cytotoxicity.[23] Inhibition of PI3Kγ signaling can promote pro-inflammatory polarization of TAMs and enhance CD8$^+$ T cell activation by activating nuclear factor κB (NF-κB) and inhibiting C/EBPβ activation.[24] Moreover, combining PI3Kα/δ inhibitors with RT and PD-1 blockade can reduce tumor growth and enhance the abscopal effect by reducing polymorphonuclear myeloid-derived suppressor cells (PMN-MDSCs), M2

Received 29 May 2025; accepted 6 November 2025;
https://doi.org/10.1016/j.omton.2025.201087.

[6]These authors contributed equally

Correspondence: In Ah Kim, MD, PhD, Department of Tumor Biology and Cancer Research Institute, Graduate School of Medicine, Seoul National University, Seoul, Republic of Korea.
E-mail: inah228@snu.ac.kr

© 2025 The Author(s). Published by Elsevier Inc. on behalf of The American Society of Gene and Cell Therapy. This is an open access article under the CC BY license (http://creativecommons.org/licenses/by/4.0/).

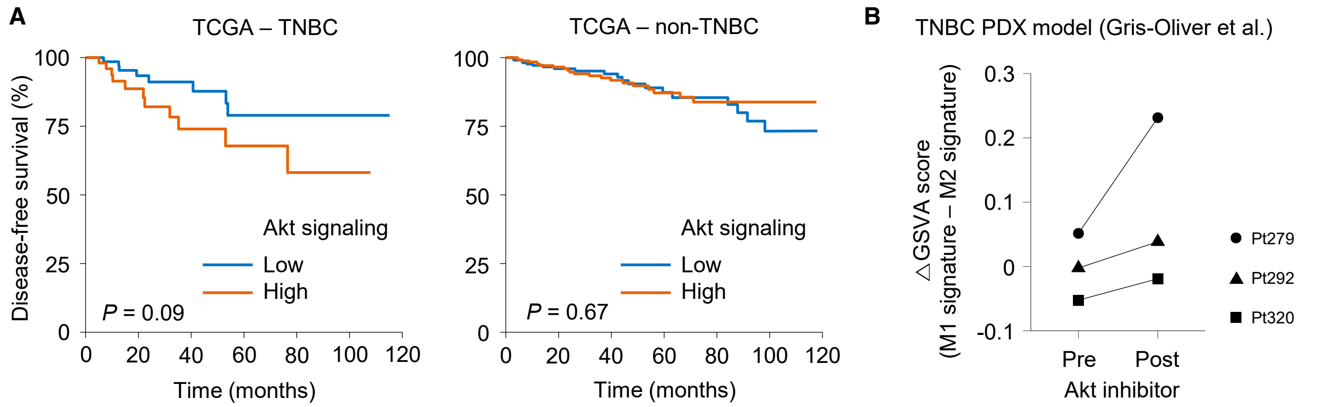

Figure 1. Impact of Akt signaling on prognosis and macrophage polarization in TNBC

(A) Kaplan-Meier disease-free survival (DFS) in TCGA cohorts stratified by Akt pathway activity (high vs. low by GSVA of an Akt-signaling signature). (B) Transcriptomic analysis of TNBC patient-derived xenograft (PDX) tumors before and after Akt-inhibitor treatment.

macrophages, and regulatory T cells (Tregs).[25] PI3Kγ/δ inhibition with PD-1 blockade in combination with RT can enhance antitumor efficacy by reducing immunosuppressive cells and increasing $CD8^+$ T cells.[26]

Given that Akt is a key downstream effector of PI3K, which plays a crucial role in immune cell modulation,[27,28] we hypothesized that Akt inhibition could alter the immunosuppressive TME and enhance ICB in combination with RT. Capivasertib was designed to suppress tumor cell survival by targeting the ATP-binding site of Akt. It has been approved for use in patients with advanced luminal breast cancer. However, its immunomodulatory role remains unclear.

Here, we show that capivasertib can reprogram bone-marrow-derived M2 macrophages toward a less immunosuppressive phenotype with enhanced pro-inflammatory characteristics. In addition, RT + αPD-L1 induced an immunosuppressive environment by decreasing M1 macrophages and increasing M2 macrophages. This immunosuppressive environment was improved by adding an Akt inhibitor, leading to partial restoration of the M1/M2 balance. Moreover, addition of Akt inhibitor to RT + αPD-L1/αCTLA-4 therapy further enhanced the anti-tumor efficacy by increasing proportion of M1/M2 ratio and $CD86^+$ DCs in a syngeneic murine TNBC model. Taken together, these results suggest that adding an Akt inhibitor to RT and ICB can increase their antitumor efficacy by modulating tumor-infiltrating myeloid cells. Such a combination could be a viable approach to overcoming therapeutic resistance of ICB.

RESULTS

Akt signaling is associated with poor prognosis and macrophage polarization in TNBC

We first explored the clinical significance of Akt signaling in breast cancer by analyzing The Cancer Genome Atlas (TCGA) database. We found that enhanced Akt signaling was associated with worse disease-free survival in TNBC patients (Figure 1A), although the difference was not statistically significant. However, this trend was not observed in non-TNBC patients, indicating that Akt signaling may exert pro-tumor role especially in TNBC. Next, we assessed the impact of Akt inhibition on the microenvironment of TNBC by analyzing RNA sequencing (RNA-seq) data acquired from patient-derived xenograft models of TNBC in the previous study.[29] Interestingly, administration of Akt inhibitor enhanced the expression of M1 signature genes relative to M2 signature genes in all three models (Figure 1B). To examine the intrinsic role of Akt signaling in macrophages, we performed small interfering RNA (siRNA)-mediated knockdown of Akt in RAW 264.7 mouse macrophage cells (Figure S1A). Akt downregulation significantly upregulated major histocompatibility complex (MHC) class II and CD86 expression in M2-polarized RAW 264.7 cells (Figure S1B). Collectively, these data suggest that Akt signaling pathway may be a therapeutic target that modulates myeloid cells in TNBC.

Akt inhibition enhances the anti-tumor efficacy of αPD-L1 in combination with RT

To evaluate the role of Akt inhibitors in combination with RT and αPD-L1, 4T1 mouse TNBC cells were inoculated into the left hindlimb and right flank of each mouse. Akt inhibitor was administered at a dose that effectively reduced pAkt levels in the primary tumor *in vivo* (Figure S2). Mice were then assigned to eight experimental groups: (1) control, (2) Akt inhibitor, (3) RT, (4) RT + Akt inhibitor, (5) αPD-L1, (6) αPD-L1 + Akt inhibitor, (7) RT + αPD-L1, and (8) triple combination (RT + αPD-L1 + Akt inhibitor). Each therapy was administered as outlined in Figure 2A. Body weight in all treatment groups was not reduced compared with the control group (Figure S3).

For the primary tumor, Akt inhibitor delayed tumor growth compared to the control group (Figure 2B). The addition of Akt inhibitor to RT further enhanced the suppression of tumor growth at the primary tumor site compared to RT alone. The triple

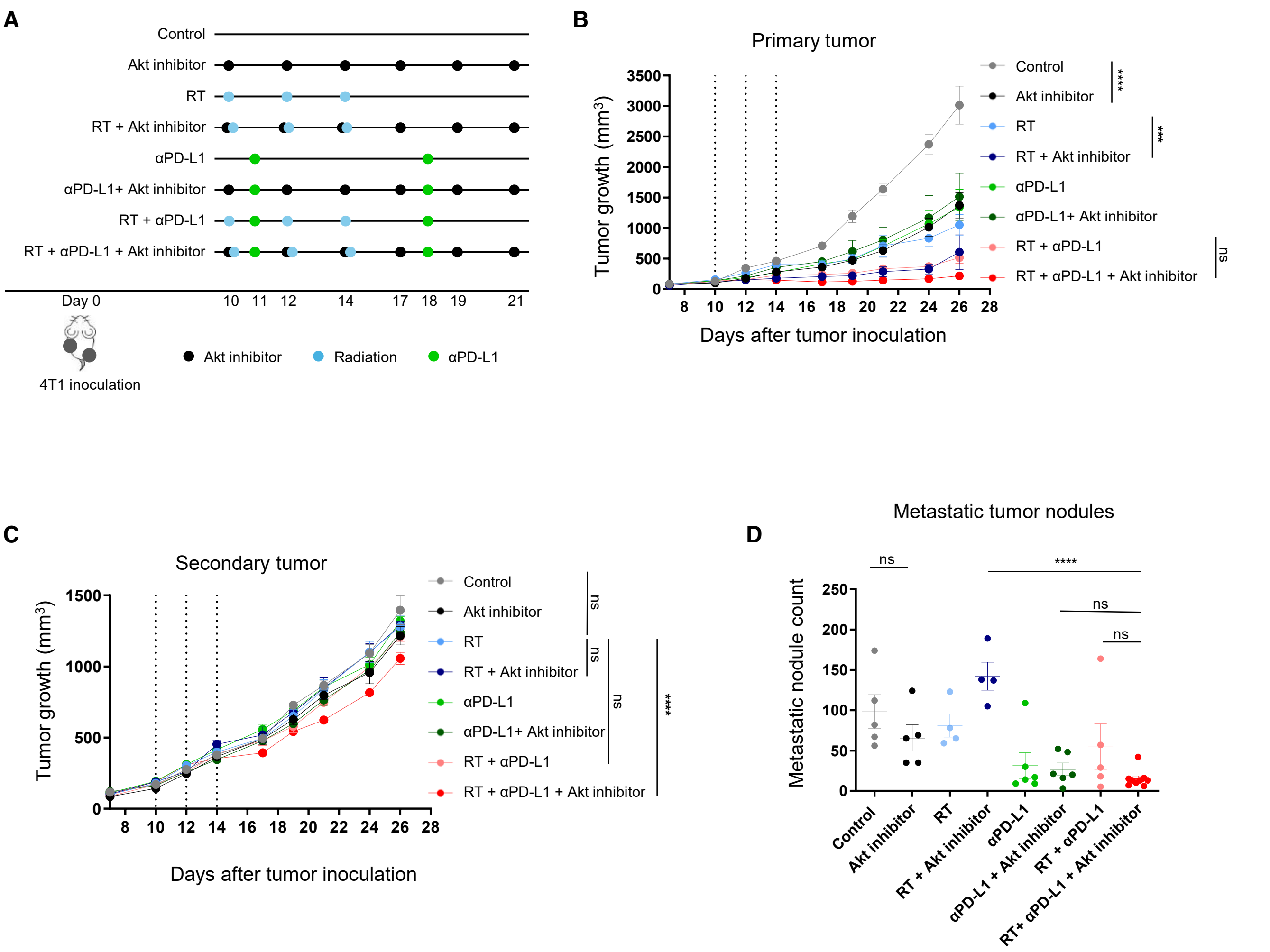

Figure 2. Antitumor efficacy of various treatments following RT, αPD-L1, and Akt inhibitor

Mouse were subcutaneously inoculated with 4T1 cells. On day 10, RT, αPD-L1, and Akt inhibitor were administered according to indicated schedule (n = 5–8 mice per group). (A) Experimental timeline of *in vivo* study. (B) Growth curve of the irradiated primary tumor. (C) Growth curve of the non-irradiated secondary tumor. The dotted lines in (B) and (C) indicate the time points of RT administration. (D) Metastatic tumor nodule count (each dot indicates an individual mouse). $*p < 0.05$, $**p < 0.01$, $***p < 0.001$, $****p < 0.0001$.

combination group showed the greatest growth delay, but the difference versus RT + αPD-L1 was not significant. RT resulted in a slight delay in the growth of secondary tumors, probably suggesting an abscopal effect (Figure 2C). While addition of either αPD-L1 or Akt inhibitor did not significantly reduce the secondary tumor growth, combined treatment with αPD-L1 and Akt inhibitor significantly inhibited the secondary tumor growth when added to RT. In line with these results, the triple combination group exhibited the lowest number of metastatic lung nodules among all treatment arms but was not significantly different from RT + αPD-L1 (Figure 2D).

Phenotypes of myeloid immune cells in the primary tumor were then analyzed. Akt inhibitor alone significantly increased the proportion of $CD86^+$ M1 macrophages and decreased $CD206^+$ M2 macrophages compared to the control (Figures 3A and 3B). We also noted that RT + αPD-L1 increased the proportion of M2 macrophages compared with control; however, the addition of the Akt inhibitor reversed this effect, restoring M2 levels to untreated levels (Figure 3B). Accordingly, the addition of Akt inhibitor to RT + αPD-L1 therapy improved the imbalanced M1/M2 ratio induced by RT + αPD-L1 (Figure 3C). We also examined whether Akt inhibitor modulated phenotypes of other myeloid cells. Akt inhibitor increased the proportion of $CD86^+$ DCs among total DCs in the primary tumor compared to the control (Figure 3D). This effect was more prominent on conventional type 2 DCs (cDC2s) than on conventional type 1 DCs (cDC1s) (Figures 3E and 3F). RT + αPD-L1 also increased mature DCs, especially cDC2s. However, Akt inhibitor did not further enhance DC maturation when it was added to RT + αPD-L1. In terms of

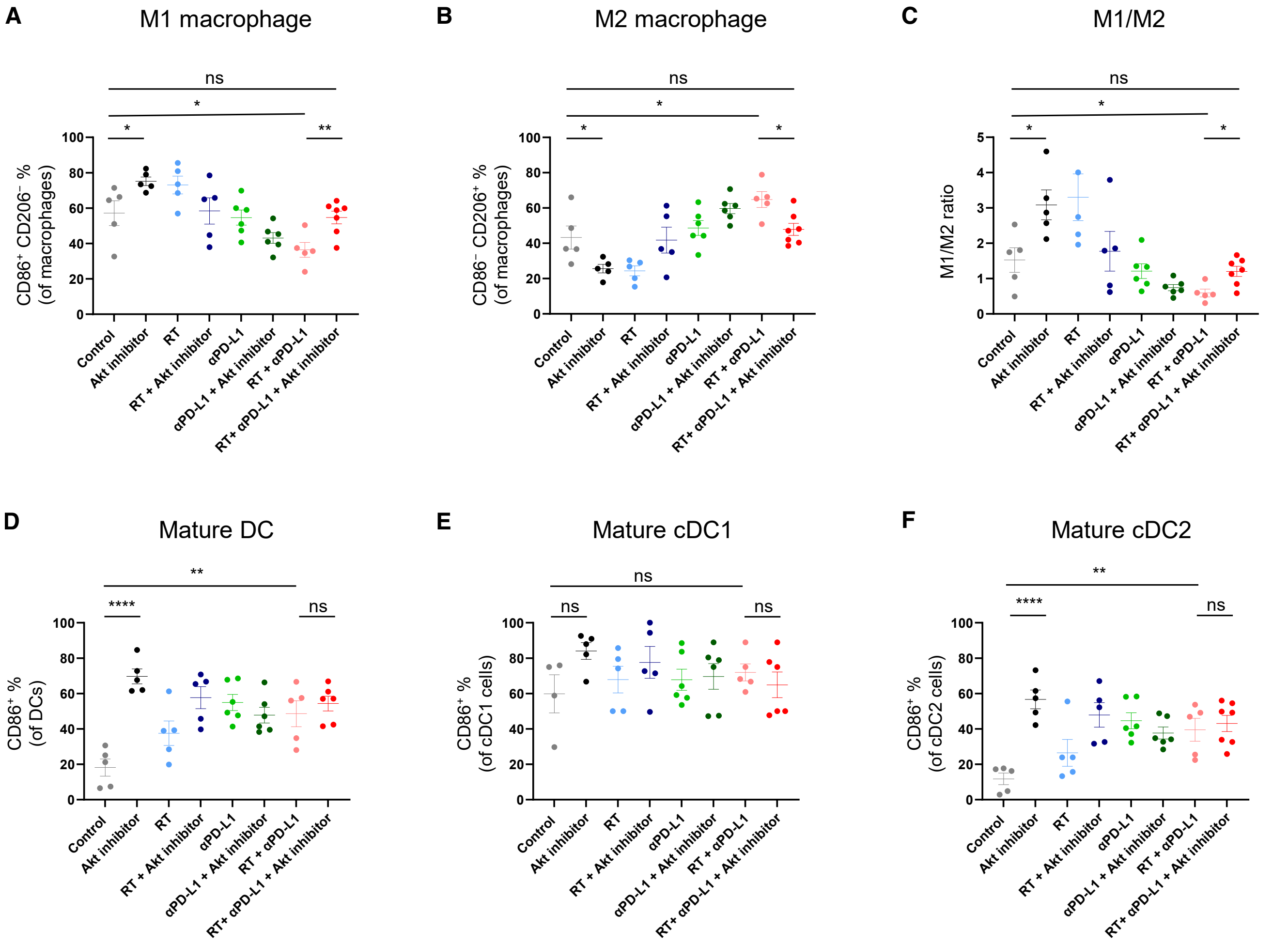

Figure 3. Myeloid immune profiling in the primary tumor following RT, αPD-L1, and Akt inhibitor

Tumor-infiltrated immune cell frequency of (A) M1 macrophages ($CD45^+$, $CD11c^-$, $CD11b^+$, $F4/80^+$, $CD206^-$, $CD86^+$); (B) M2 macrophages ($CD45^+$, $CD11c^-$, $CD11b^+$, $F4/80^+$, $CD206^+$, $CD86^-$); (C) M1-to-M2 ratio; (D) mature dendritic cells ($CD45^+$, $CD11c^+$, $CD86^+$); (E) mature cDC1 ($CD45^+$, $CD11c^+$, $CD103^+$, $CD11b^-$, $CD86^+$); and (F) mature cDC2 ($CD45^+$, $CD11c^+$, $CD103^-$, $CD11b^+$, $CD86^+$). All data represent the mean ± SEM (each dot indicates individual mice; $n = 5–7$ mice per group). *$p < 0.05$, **$p < 0.01$, ***$p < 0.001$, ****$p < 0.0001$.

MDSCs, Akt inhibitor alone did not alter the proportion of M-MDSCs or PMN-MDSCs (Figures S4A and S4B). However, the addition of Akt inhibitor to RT + αPD-L1 significantly decreased M-MDSCs without affecting PMN-MDSCs. These results suggest that Akt inhibitor may shift the TME to a less immunosuppressive state by modulating tumor-infiltrating myeloid cells, especially when it is added to RT + αPD-L1.

Akt inhibitor enhances the anti-tumor effect of αPD-L1/αCTLA-4 in combination with RT

Next, we assessed whether the Akt inhibitor could improve the anti-tumor effects of RT or a combined blockade of PD-L1 and CTLA-4 (αPD-L1/αCTLA-4). We evaluated the following experimental groups: (1) control, (2) Akt inhibitor, (3) RT, (4) RT + Akt inhibitor, (5) αPD-L1/αCTLA-4, (6) αPD-L1/αCTLA-4 + Akt inhibitor, (7) RT + αPD-L1/αCTLA-4, and (8) quadruple combination (RT + αPD-L1/αCTLA-4 + Akt inhibitor). Body weight in all treatment groups was not reduced compared with the control group (Figure S5). Quadruple combination inhibited the growth of primary tumors more significantly than any treatment schemes (Figure 4A). For secondary tumor, addition of Akt inhibitor to RT + αPD-L1/αCTLA-4 resulted in greater tumor growth inhibition than RT + αPD-L1/αCTLA-4, although the difference was not statistically significant (Figure 4B). Importantly, the number of metastatic lung nodules was reduced by adding Akt inhibitor to RT + αPD-L1/αCTLA-4 (Figures 4C and 4D), suggesting that Akt inhibitor contributed to enhanced local and systemic anti-tumor responses even when it was added to RT + αPD-L1/αCTLA-4. Given that RT has been reported to modulate PI3K/AKT signaling and suppress epithelial-mesenchymal transition (EMT),[30] we assessed αSMA,

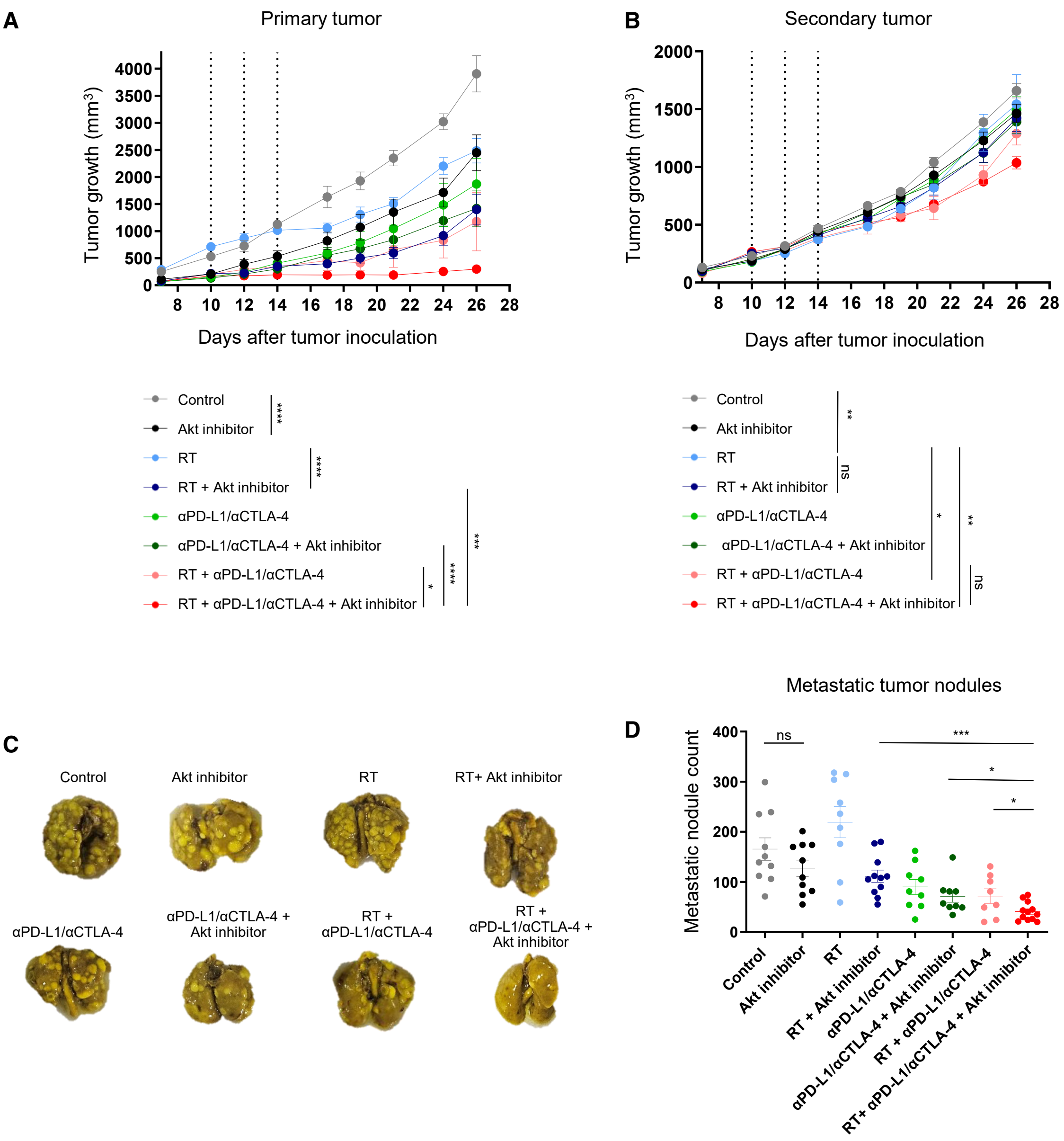

Figure 4. Antitumor efficacy of various treatments following RT, αPD-L1/αCTLA-4, and Akt inhibitor

(A) Primary tumor growth curve and (B) secondary tumor growth curve. The dotted lines in (A) and (B) indicate the time points of RT administration. (C) Lung image on day 28 after tumor inoculation. (D) Metastatic tumor nodule count (each dot indicates an individual mouse). Tumor growth curves (A, B) are shown from one representative experiment (*n* = 5 mice per group). Metastasis data (D) are pooled from independent experiments (*n* = 8–12 mice per group). All data represent the mean ± SEM. **p* < 0.05, ***p* < 0.01, ****p* < 0.001, *****p* < 0.0001.

vimentin, and E-cadherin in the primary tumor. Akt inhibitor or RT alone suppressed EMT (Figures S6A–S6C). Adding the Akt inhibitor to RT further enhanced this suppression, and the quadruple regimen showed the strongest suppression effect. In addition, co-localization of αSMA^+ pericytes and $CD31^+$ endothelial cells increased with the Akt inhibitor (Figures S7A and S7B), suggesting the vasculature normalization by Akt inhibitor. Importantly, adding Akt inhibitor to RT + αPD-L1/αCTLA-4 also increased the co-localization. These data show that adding Akt inhibitor to RT + αPD-L1/αCTLA-4 enhances anti-tumor efficacy, accompanied by reduced EMT and normalized tumor vasculature.

Akt inhibitor alleviates the immunosuppressive phenotype of macrophages

We next evaluated phenotypes of macrophages according to treatments. Importantly, the addition of Akt inhibitor to the

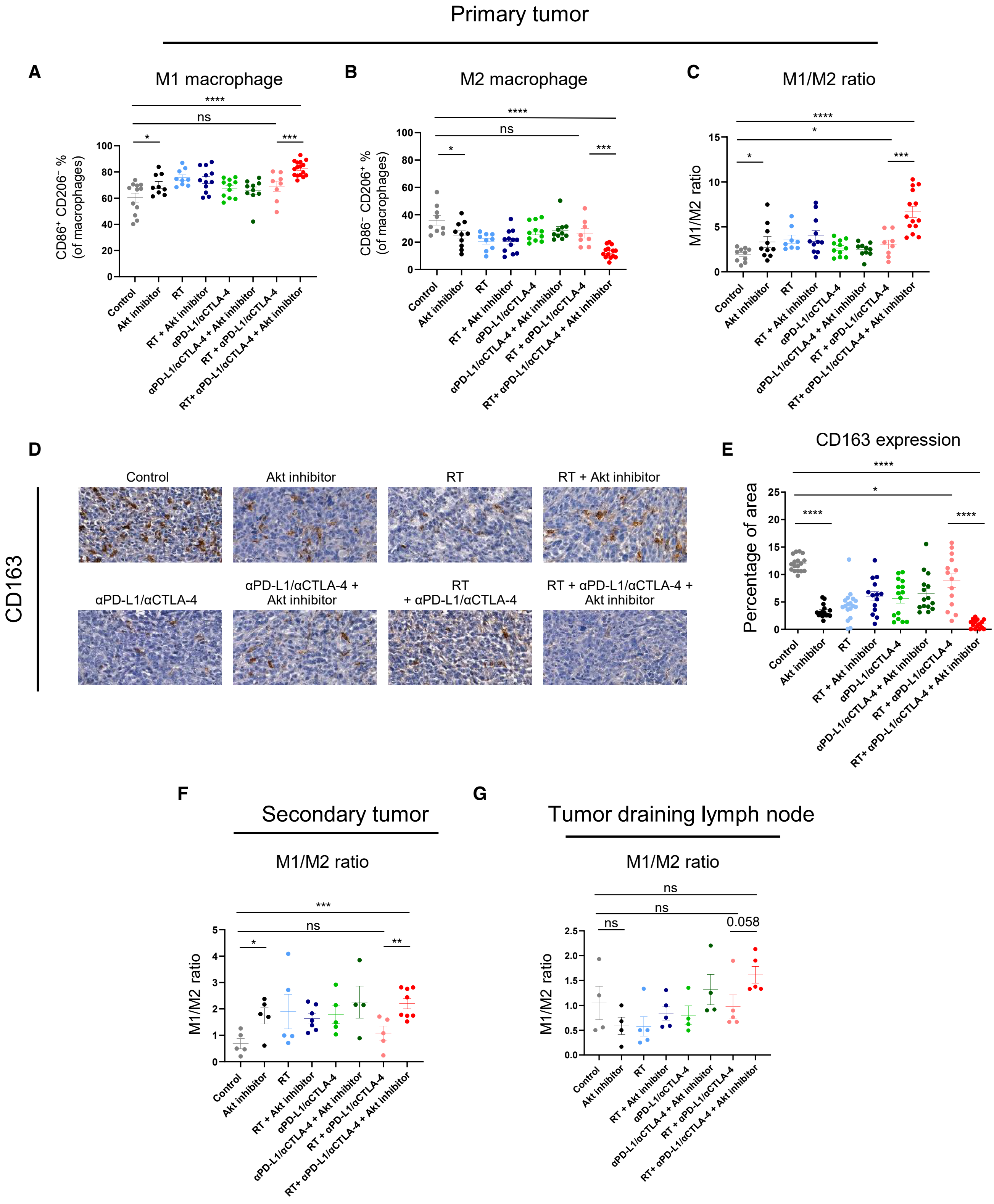

(legend on next page)

RT + αPD-L1/αCTLA-4 treatment group increased M1 macrophages and decreased M2 macrophages (Figures 5A and 5B), leading to a higher M1/M2 ratio (Figure 5C). Decrease of M2 macrophages within the tumor by Akt inhibitor, even when combined with RT + αPD-L1/αCTLA-4, was further supported by a reduction in CD163 expression in immunohistochemistry (IHC) staining (Figures 5D and 5E). Notably, while Akt inhibitor induced only partial improvement in M1/M2 balance when it was added to the RT + αPD-L1 therapy, its combination with RT + αPD-L1/αCTLA-4 resulted in a potent reprogramming effect, markedly elevating M1 macrophage levels and reducing M2 macrophages. This suggests a synergistic enhancement of macrophage polarization by the quadruple combination. Such increase of the M1/M2 ratio by the addition of Akt inhibitor was also observed in the secondary tumor (Figure 5F) and tumor-draining lymph nodes (Figure 5G), suggesting systemic effects of Akt inhibitor on modulating macrophages. Corresponding M1 and M2 macrophage populations are shown in Figures S8A–S8D.

We next assessed direct effects of Akt inhibitor on M1- and M2-polarized macrophages. In M1-polarized macrophages, treatment with 1 μM Akt inhibitor increased the $CD86^+/CD206^+$ ratio, whereas higher doses showed less prominent effects (Figures 6A and 6B). Additionally, in M2-polarized macrophages, Akt inhibitor treatment increased the $CD86^+/CD206^+$ ratio at higher doses (Figures 6C and 6D). These results demonstrate a direct, cell-intrinsic effect of Akt inhibition that reprograms macrophage polarization toward an M1-like phenotype.

Akt inhibitor enhances DC maturation and reduces M-MDSCs

We also assessed DC maturation following RT, αPD-L1/αCTLA-4, and Akt inhibition. Notably, RT + αPD-L1/αCTLA-4 + Akt inhibitor significantly increased $CD86^+$ DCs in primary tumors compared to RT + αPD-L1/αCTLA-4 (Figure 7A). When we analyzed CD86 expression among conventional DC type 1 and type 2, the addition of Akt inhibitor to RT + αPD-L1/αCTLA-4 significantly increased maturation in both cDC1 and cDC2 (Figures 7B and 7C). However, the addition of Akt inhibitor did not alter mature DC subsets, including cDC1 and cDC2, in the secondary tumor or spleen (Figures S9A–S9F). These results suggest that adding Akt inhibitor can enhance cDC1 and cDC2 maturation, which may lead to increased presentation of tumor antigens in primary tumors. We also found that when Akt inhibitor was added to RT + αPD-L1/αCTLA-4, M-MDSC proportion was significantly decreased, while no reduction was observed in PMN-MDSCs (Figures S10A and S10B).

DISCUSSION

While ICBs such as αPD-L1 have shown promising results in many solid tumors, their efficacy in immunologically cold tumors, such as TNBC, remains limited due to a highly immune-suppressive TME.[31,32] To overcome this limitation, combining RT, which can induce immunogenic cell death and enhance tumor immunogenicity, with ICBs has emerged as a promising therapeutic option.[3–6] In this study, we demonstrated that anti-tumor effects of RT + ICB therapy were enhanced by the addition of Akt inhibitors in a murine TNBC model. To the best of our knowledge, this is the first study demonstrating the efficacy of a triple combination therapy involving RT, ICB, and an Akt inhibitor.

Akt, a key component of the PI3K/Akt/mTOR pathway, can regulate tumor cell survival, proliferation, and metastasis. While Akt inhibitors such as MK-2206 and capivasertib (AZD5363) have shown moderate anti-tumor activities in cancers including prostate cancer, melanoma, and breast cancer, their clinical efficacy has often been limited, especially when they are used as monotherapy or when they are combined with chemotherapy.[33,34]

Capivasertib, an Akt inhibitor used in this study, was recently approved by the Food and Drug Administration (FDA) in combination with fulvestrant for HR+/HER2– breast cancer patients. However, in TNBC, its therapeutic effect has been less encouraging. For instance, a phase III CAPItello-290 trial showed that combining capivasertib with paclitaxel did not improve overall survival, highlighting the need for alternative combination strategies to enhance anti-tumor responses of this aggressive tumor type.

In this regard, our study proposed that adding capivasertib with RT and ICB might represent a promising approach. By simultaneously targeting tumor-intrinsic survival pathways and promoting immune-mediated anti-tumor responses, this triple combination has the potential to overcome some limitations observed with conventional Akt-inhibitor-based regimens in TNBC. In line with this, our study demonstrated that Akt inhibition could exert an immunomodulatory effect within the TNBC TME, particularly when combined with RT and ICB.

The M1/M2 ratio is closely associated with tumor progression. A decrease in M2 macrophage infiltration has been linked to improved survival in various cancer types.[35,36] The Akt inhibitor alone significantly reduced tumor growth in our model, increased the proportion of $CD86^+$ M1 macrophages, decreased M2 macrophages, and elevated CD86 expression on DCs. The observed suppression of M2 macrophages is consistent with previous reports showing that PI3K/Akt/mTOR signaling is essential for interleukin (IL)-4-driven M2 macrophage polarization and function.[27,37] Based on these findings, we next examined whether Akt inhibition could modulate the immune landscape when combined with RT and αPD-L1.

Figure 5. Macrophage profiling in the tumor microenvironment following RT, αPD-L1/αCTLA-4, and Akt inhibitor

Primary-tumor-infiltrated immune cell frequency of (A) M1 macrophages, (B) M2 macrophages, and (C) M1-to-M2 ratio. (D) IHC staining for CD163 in primary tumor sections; scale bars, 20 μm. (E) Quantification of CD163 IHC staining results. M1-to-M2 ratio in (F) secondary tumor and (G) tumor-draining lymph node. All data represent the mean ± SEM. For (A–C, F, G), each dot indicates individual mice (n = 8–16). For (E), n indicates the number of IHC images analyzed (n = 14–18). *$p < 0.05$, **$p < 0.01$, ***$p < 0.001$, ****$p < 0.0001$.

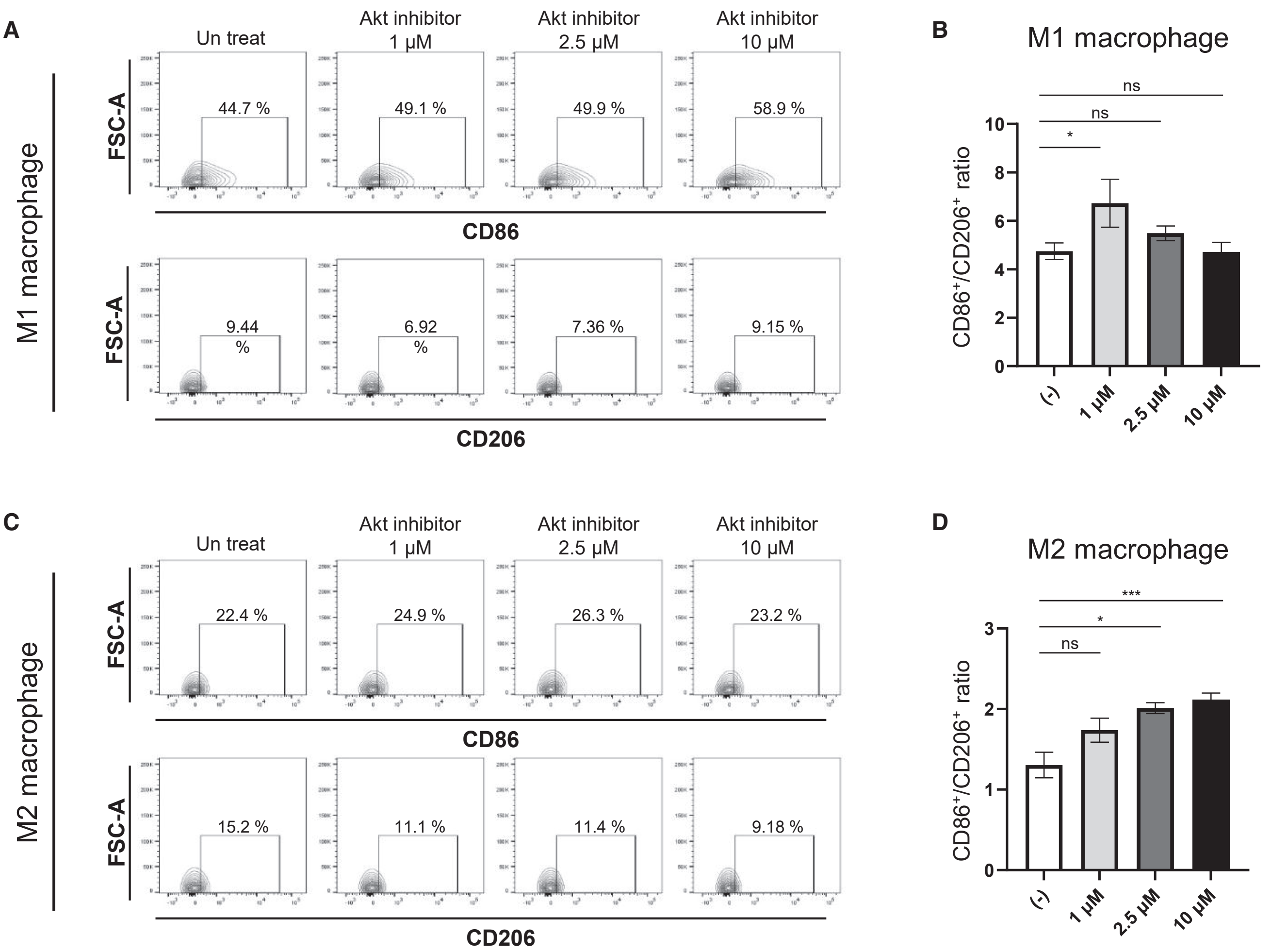

Figure 6. CD86 and CD206 expression in BMDM-derived M1/M2 macrophages following Akt inhibitor treatment

BMDMs were polarized into M1 (LPS + IFN-γ) or M2 (IL-4) macrophages and treated with Akt inhibitor at indicated concentrations. (A) Representative FACS plots showing CD86 and CD206 expression in M1 macrophages. (B) Quantification of the $CD86^+/CD206^+$ ratio in M1 macrophages. (C) Representative FACS plots showing CD86 and CD206 expression in M2 macrophages. (D) Quantification of the $CD86^+/CD206^+$ ratio in M2 macrophages. Positive gating for CD86 and CD206 were determined based on M0 macrophages (negative control) with a 5% threshold. All data represent the mean ± SEM (n = 3–8). *$p < 0.05$, **$p < 0.01$, ***$p < 0.001$.

Interestingly, although RT combined with αPD-L1 reduced tumor burden, it paradoxically increased the infiltration of M2 macrophages in our model. The addition of an Akt inhibitor effectively reversed this effect and reduced M2 macrophage accumulation. In addition, in a separate experiment using dual ICB (αPD-L1/αCTLA-4), M2 accumulation was less pronounced due to the potential effect of αCTLA-4. Still, the addition of Akt inhibition further reduced M2 macrophage levels, suggesting its robust and consistent capacity to modulate the immunosuppressive myeloid landscape. This effect was also observed in tumor-draining lymph nodes and secondary tumors, suggesting a systemic anti-tumor effect of Akt inhibition. However, despite these immunologic changes, suppression of secondary tumor growth was less evident, whereas a reduction in lung metastases was observed, suggesting that systemic immune modulation may differentially impact disseminated versus established tumor sites. Although the addition of the Akt inhibitor to RT + αPD-L1 did not further enhance DC maturation, its combination with RT + αPD-L1/αCTLA-4 led to a marked increase in $CD86^+$ DCs within the TME. This effect was observed in both cDC1 and cDC2 subsets, suggesting enhanced antigen-presenting capacity.

Despite these promising findings, this study has several limitations. First, the use of a single murine TNBC model might not fully capture the heterogeneity of breast cancer or other immunologically cold tumors. Future studies should validate this combination strategy in additional tumor models, including other TNBC cell lines, as well as pancreatic and prostate cancers. Second, given that the combination of RT, ICB, and Akt inhibition involves multiple layers of immune modulation, there is a potential risk of immune overactivation or autoimmune responses, underscoring the need for

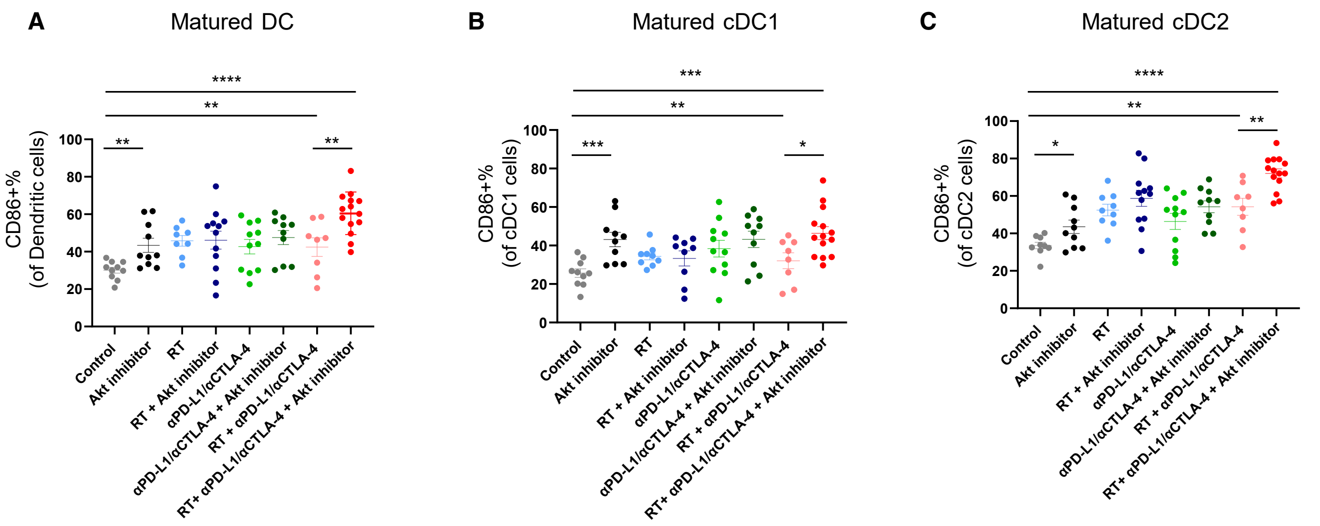

Figure 7. Proportion of mature DCs (cDC1 and cDC2) in primary tumors following RT, αPD-L1/αCTLA-4, and Akt inhibitor

Tumor-infiltrated immune cell frequency of (A) matured dendritic cells, (B) matured cDC1, and (C) matured cDC2. All data represent the mean ± SEM (n = 8–14). *$p < 0.05$, **$p < 0.01$, ***$p < 0.001$, ****$p < 0.0001$. Park and colleagues demonstrate that Akt inhibition enhances the efficacy of radiotherapy and immune checkpoint blockade in triple-negative breast cancer by modulating myeloid cells. These findings support combination strategies targeting Akt to overcome resistance in immune-cold tumors.

thorough safety evaluations. Moreover, we did not analyze the immunologic changes in metastatic lung nodules. Although we characterized the systemic effects of Akt inhibition alone or in combination with RT and ICB by analyzing secondary tumors, evaluation of metastatic foci would be helpful to gain insight into the systemic modulation of myeloid cells in the context of Akt inhibition. Lastly, our mechanistic analyses were primarily focused on myeloid cells such as macrophages and DCs. To gain a more comprehensive understanding of the immune response, further studies should evaluate effects of this combination therapy on other lymphocyte populations, including $CD8^+$ and $CD4^+$ T cells, as well as regulatory T cells.

Taken together, these findings provide mechanistic insight into how Akt inhibition can synergize with RT and ICB by reprogramming the immunosuppressive myeloid compartment and fostering a more inflamed immune milieu. In conclusion, Akt inhibition could be a viable strategy to overcome therapeutic resistance of RT and ICB in immunologically cold tumors, such as breast cancer.

MATERIALS AND METHODS

Analysis of public RNA-seq database

RNA-seq data and clinical information of breast cancer in TCGA database was downloaded from the Broad Institute GDAC Firehose (https://gdac.broadinstitute.org). Additionally, raw data from bulk RNA-seq of tumor tissues collected before and after administration of an Akt inhibitor in patient-derived xenograft models from three TNBC patients were downloaded from the Gene Expression Omnibus (GSE114794).[29] Expressions of Akt signaling pathway signature,[38] as well as gene signatures for M1 and M2 macrophages,[39] were analyzed using gene set variation analysis.

Cell line preparation

The 4T1 murine triple-negative breast cancer cell line was obtained from the Japanese Collection of Research Bioresources (JCRB) Cell Bank (Osaka, Japan). The RAW 264.7 murine macrophage cell line was obtained from the Korean Cell Line Bank (Seoul, Korea). Cells were cultured in RPMI-1640 medium or DMEM medium (Welgene, Korea) supplemented with 10% fetal bovine serum (FBS; Gibco, USA) and 1% penicillin-streptomycin (Welgene, Korea). These cells were maintained at 37°C in a 5% CO_2 humidified incubator.

siRNA experiment

RAW 264.7 cells were polarized into M2 macrophages by stimulation with IL-4 (20 ng/mL; PeproTech, USA, 204-14) for 24–48 h. Cells were then transfected with control siRNA (Cell Signaling Technology, USA, 6568s) or Akt-specific siRNA (Cell Signaling Technology, USA, 6211s) for 36 h. After treatment, cells were harvested and prepared for flow cytometry or western blot analysis. For western blotting, total cellular proteins were extracted using RIPA buffer supplemented with protease and phosphatase inhibitors (Cell Signaling Technology, USA, 5872s), quantified by BCA assay, and separated by SDS–PAGE. Proteins were transferred onto PVDF membranes, blocked with 5% BSA in Tris-buffered saline containing 0.1% Tween 20 (TBS-T), and probed with primary antibodies against Akt (Cell Signaling Technology, USA, 4691T) and β-actin (Cell Signaling Technology, USA, 122262s), followed by HRP-conjugated secondary antibodies. Signals were detected using an enhanced chemiluminescence (ECL) system.

Animal model and experimental design

Five- to six-week-old female Balb/c wild-type mice were purchased from Orient Bio (Seongnam, Korea). 4T1 cells were subcutaneously

inoculated into the right hindlimb (6×10^5 cells) and left flank (1×10^5 cells) of each mouse on day 0. After 1 week, mice were randomly grouped into two experimental sets, each consisting of eight groups ($n \geq 5$ per group). The first set included control, Akt inhibitor, RT, RT + Akt inhibitor, αPD-L1, αPD-L1 + Akt inhibitor, RT + αPD-L1, and RT + αPD-L1 + Akt inhibitor. The second set included control, Akt inhibitor, RT, RT + Akt inhibitor, αPD-L1/αCTLA-4, αPD-L1/αCTLA-4 + Akt inhibitor, RT + αPD-L1/αCTLA-4, and RT + αPD-L1/αCTLA-4 + Akt inhibitor. The RT-containing group was irradiated with 8 Gy in three fractions every other day for 1 week using X-Rad 320 (Precision X-Ray, USA). Only primary tumor sites (right hindlimb) were exposed, while the rest of the body was shielded from radiation. Mice were anesthetized with 40% Alfaxan Multidose and 20% Rompun (intraperitoneally [i.p.]) prior to irradiation. Capivasertib (150 mg/kg; AstraZeneca, UK) was administered via oral gavage on days 10, 12, 14, 17, 19, and 21. Mice in the αPD-L1 group (5 mg/kg; AstraZeneca) or the αPD-L1/αCTLA-4 combination group (5 mg/kg each; AstraZeneca) received intraperitoneal injections on days 11 and 18. Tumor volumes were measured every other day using a caliper. It was calculated using the formula of $0.52 \times \text{length} \times \text{width}^2$. On day 28, mice were sacrificed and their lung, spleen, tumor tissues were isolated. All mice were maintained according to Institutional Animal Care and Use Committee (IACUC) guidelines of Seoul National University Bundang Hospital (# BA-2112-334-009) following animal ethics rules.

Single cell preparation

Tumor tissues were minced and incubated with collagenase IV (100 U/mL; Gibco, USA, 17104019) and DNase I (0.2 mg/mL; Roche, Switzerland, 1010415900) at 37°C for 30 min. After incubation, samples were filtered using a 70 μm strainer and washed with PBS. For splenocyte isolation, spleens were mashed through strainer, lysed with ACK lysis buffer (Gibco, USA, A1049201), and washed with PBS. Lymph nodes were mashed through a 70 μm strainer. All single-cell suspensions were then prepared for flow cytometry analysis.

Flow cytometry analysis

Cells were stained with a LIVE/DEAD Fixable Red Dead Cell Stain Kit (Thermo Fisher Scientific, USA, L34972) to exclude dead cells, followed by Fc receptor blocking with TruStain FcX PLUS (anti-mouse CD16/32; BioLegend, USA, 156604). Cells were then incubated with fluorochrome-conjugated antibodies targeting MHC II, CD11c, CD45, Ly6G, CD86, CD11b, Ly6C, CD103, CD206, and F4/80 for 15 min at room temperature, followed by washing with PBS. Fluorescent antibodies were obtained from BioLegend (MHC II, 107643; CD11c, 117330; CD11b, 101205; CD103, 121425; CD206, 141708; F4/80, 123117) or BD Biosciences (CD45, 563891; Ly6G, 563005; CD86, 740877; Ly6C, 128012). Samples were analyzed using a BD FACSymphony A1 flow cytometer (BD Biosciences, USA). Data were processed using FlowJo v.10.8.1 (BD Biosciences, USA).

Immunohistochemistry staining

Mouse tissues were fixed with 4% paraformaldehyde (PFA; T&I, Korea; BPP-9004), embedded in paraffin, and sectioned at a thickness of 5 μm. Sections were then mounted on slides, followed by deparaffinization and rehydration. Antigen retrieval was performed using Tris-EDTA (pH 9.0) or citrate buffer (pH 6.0) at 100°C for 30 min. Slides were blocked with 3% BSA and incubated overnight at 4°C with anti-CD163 antibody (Abcam, UK; ab182422); anti-pAkt (Ser473) antibody (Cell Signaling Technology, USA; 4060s); anti-αSMA (Cell Signaling Technology, USA; 19245T); anti-E-cadherin (Cell Signaling Technology, USA; 3195T); and anti-Vimentin (Cell Signaling Technology, USA; 5741T). After washing with 0.1% phosphate-buffered saline with Tween 20 (PBS-T), slides were then incubated with HRP-conjugated secondary anti-rabbit antibodies at room temperature for 30 min. Detection was performed using a Dako REAL EnVision Detection System (Dako, Denmark, K3468). Subsequently, slides were counterstained with hematoxylin, mounted with an antifade reagent, and scanned using a slide scanner (3DHISTECH, Budapest, Hungary). Images were captured using CaseViewer software (v.2.4) and quantified using ImageJ software (NIH, USA).

Immunofluorescence staining

Paraffin-embedded tissue slides were deparaffinized and subjected to antigen retrieval in citrate buffer (10 mM sodium citrate, pH 6.0), as described above for IHC. Slides were then blocked and incubated overnight at 4°C with FITC-labeled anti-CD31 antibody (Thermo Fisher Scientific, USA, 11-0311-82) and anti-αSMA primary antibody (Cell Signaling Technology, USA; 19245T). After washing with 0.05% PBS-T, slides were incubated with Alexa-Fluor-594-conjugated anti-rabbit secondary antibody (Thermo Fisher Scientific, USA, A-21209). Finally, slides were mounted and imaged using a confocal microscope (LSM 800, Zeiss, Germany).

In vitro macrophage reprogramming assay

Bone-marrow-derived macrophages (BMDMs) were isolated from mouse femurs and cultured with macrophage colony-stimulating factor (M-CSF; 20 ng/mL; PeproTech, USA, 315-02) for 7 days to promote macrophage differentiation. BMDMs were differentiated into M1 and M2 macrophages using lipopolysaccharide (LPS; 100 ng/mL; Sigma-Aldrich, USA, L6529-1MG) and interferon-γ (IFN-γ; 20 ng/mL; R&D Systems, USA, 485-MI-100/CF), or IL-4 (20 ng/mL; PeproTech, 204-14), respectively, for 24–48 h. Akt inhibitor at different concentrations (0.1, 1, 10, and 100 μM) was incubated with BMDMs for 24 h. CD86 and CD206 expression levels were then analyzed by flow cytometry analysis.

Statistical analysis

Kaplan-Meier curves were compared using the Cox proportional hazards model. Unpaired Student's t test was applied to compare continuous variables. Two-way analysis of variance (ANOVA) with Tukey's multiple comparisons test was used for analyzing growth curves. All data are presented as mean ± standard error of the mean (SEM). Data were analyzed using GraphPad Prism 9 (GraphPad Software, USA). p value <0.05 was considered statistically significant.

DATA AND CODE AVAILABILITY

The data supporting the findings of this study are available from the corresponding author upon reasonable request.

ACKNOWLEDGMENTS

This work was supported by grants from KHIDI & AstraZeneca and the National Research Foundation of Korea #RS-2023-NR077241. The graphical abstract was created using BioRender.com.

AUTHOR CONTRIBUTIONS

N.P. performed the experiments, carried out data analysis, and wrote the manuscript. S.H.J. supervised the experimental work and provided conceptual input. Y.K. and S.K. assisted with flow cytometry data interpretation and repeated key experiments. I.A.K. supervised the overall study, secured funding, and proposed the research direction.

DECLARATION OF INTERESTS

I.A.K. received research support from AstraZeneca in the form of study drugs (capivasertib, α-PD-L1, and α-CTLA-4 antibodies) used in this study.

SUPPLEMENTAL INFORMATION

Supplemental information can be found online at https://doi.org/10.1016/j.omton.2025.201087.

REFERENCES

1. Braun, D.A., Burke, K.P., and Van Allen, E.M. (2016). Genomic Approaches to Understanding Response and Resistance to Immunotherapy. Clin. Cancer Res. *22*, 5642–5650. https://doi.org/10.1158/1078-0432.Ccr-16-0066.

2. Hanna, A., and Balko, J.M. (2021). Breast cancer resistance mechanisms: challenges to immunotherapy. Breast Cancer Res. Treat. *190*, 5–17. https://doi.org/10.1007/s10549-021-06337-x.

3. Zeng, J., See, A.P., Phallen, J., Jackson, C.M., Belcaid, Z., Ruzevick, J., Durham, N., Meyer, C., Harris, T.J., Albesiano, E., et al. (2013). Anti-PD-1 blockade and stereotactic radiation produce long-term survival in mice with intracranial gliomas. Int. J. Radiat. Oncol. Biol. Phys. *86*, 343–349. https://doi.org/10.1016/j.ijrobp.2012.12.025.

4. Dovedi, S.J., Adlard, A.L., Lipowska-Bhalla, G., McKenna, C., Jones, S., Cheadle, E.J., Stratford, I.J., Poon, E., Morrow, M., Stewart, R., et al. (2014). Acquired resistance to fractionated radiotherapy can be overcome by concurrent PD-L1 blockade. Cancer Res. *74*, 5458–5468. https://doi.org/10.1158/0008-5472.Can-14-1258.

5. Gong, X., Li, X., Jiang, T., Xie, H., Zhu, Z., Zhou, F., and Zhou, C. (2017). Combined Radiotherapy and Anti-PD-L1 Antibody Synergistically Enhances Antitumor Effect in Non-Small Cell Lung Cancer. J. Thorac. Oncol. *12*, 1085–1097. https://doi.org/10.1016/j.jtho.2017.04.014.

6. Theelen, W.S.M.E., Chen, D., Verma, V., Hobbs, B.P., Peulen, H.M.U., Aerts, J.G.J.V., Bahce, I., Niemeijer, A.L.N., Chang, J.Y., de Groot, P.M., et al. (2021). Pembrolizumab with or without radiotherapy for metastatic non-small-cell lung cancer: a pooled analysis of two randomised trials. Lancet Respir. Med. *9*, 467–475. https://doi.org/10.1016/s2213-2600(20)30391-x.

7. Akkari, L., Bowman, R.L., Tessier, J., Klemm, F., Handgraaf, S.M., de Groot, M., Quail, D.F., Tillard, L., Gadiot, J., Huse, J.T., et al. (2020). Dynamic changes in glioma macrophage populations after radiotherapy reveal CSF-1R inhibition as a strategy to overcome resistance. Sci. Transl. Med. *12*, eaaw7843. https://doi.org/10.1126/scitranslmed.aaw7843.

8. Qian, B.Z., and Pollard, J.W. (2010). Macrophage diversity enhances tumor progression and metastasis. Cell *141*, 39–51. https://doi.org/10.1016/j.cell.2010.03.014.

9. Wang, S., Wang, J., Chen, Z., Luo, J., Guo, W., Sun, L., and Lin, L. (2024). Targeting M2-like tumor-associated macrophages is a potential therapeutic approach to overcome antitumor drug resistance. npj Precis. Oncol. *8*, 31. https://doi.org/10.1038/s41698-024-00522-z.

10. Sica, A., and Mantovani, A. (2012). Macrophage plasticity and polarization: in vivo veritas. J. Clin. Investig. *122*, 787–795. https://doi.org/10.1172/jci59643.

11. Dehne, N., Mora, J., Namgaladze, D., Weigert, A., and Brüne, B. (2017). Cancer cell and macrophage cross-talk in the tumor microenvironment. Curr. Opin. Pharmacol. *35*, 12–19. https://doi.org/10.1016/j.coph.2017.04.007.

12. Sica, A., Schioppa, T., Mantovani, A., and Allavena, P. (2006). Tumour-associated macrophages are a distinct M2 polarised population promoting tumour progression: potential targets of anti-cancer therapy. Eur. J. Cancer *42*, 717–727. https://doi.org/10.1016/j.ejca.2006.01.003.

13. Khan, S.U., Khan, M.U., Azhar Ud Din, M., Khan, I.M., Khan, M.I., Bungau, S., and Hassan, S.S.U. (2023). Reprogramming tumor-associated macrophages as a unique approach to target tumor immunotherapy. Front. Immunol. *14*, 1166487. https://doi.org/10.3389/fimmu.2023.1166487.

14. Yang, L., Dong, Y., Li, Y., Wang, D., Liu, S., Wang, D., Gao, Q., Ji, S., Chen, X., Lei, Q., et al. (2019). IL-10 derived from M2 macrophage promotes cancer stemness via JAK1/STAT1/NF-κB/Notch1 pathway in non-small cell lung cancer. Int. J. Cancer *145*, 1099–1110. https://doi.org/10.1002/ijc.32151.

15. Chen, Y.J., Li, G.N., Li, X.J., Wei, L.X., Fu, M.J., Cheng, Z.L., Yang, Z., Zhu, G.Q., Wang, X.D., Zhang, C., et al. (2023). Targeting IRG1 reverses the immunosuppressive function of tumor-associated macrophages and enhances cancer immunotherapy. Sci. Adv. *9*, eadg0654. https://doi.org/10.1126/sciadv.adg0654.

16. Wei, Y.T., Wang, X.R., Yan, C., Huang, F., Zhang, Y., Liu, X., Wen, Z.F., Sun, X.T., Zhang, Y., Chen, Y.Q., et al. (2022). Thymosin α-1 Reverses M2 Polarization of Tumor-Associated Macrophages during Efferocytosis. Cancer Res. *82*, 1991–2002. https://doi.org/10.1158/0008-5472.Can-21-4260.

17. Rohila, D., Park, I.H., Pham, T.V., Jones, R., Tapia, E., Liu, K.X., Tamayo, P., Yu, A., Sharabi, A.B., and Joshi, S. (2023). Targeting macrophage Syk enhances responses to immune checkpoint blockade and radiotherapy in high-risk neuroblastoma. Front. Immunol. *14*, 1148317. https://doi.org/10.3389/fimmu.2023.1148317.

18. Li, K., Shi, H., Zhang, B., Ou, X., Ma, Q., Chen, Y., Shu, P., Li, D., and Wang, Y. (2021). Myeloid-derived suppressor cells as immunosuppressive regulators and therapeutic targets in cancer. Signal Transduct. Target. Ther. *6*, 362. https://doi.org/10.1038/s41392-021-00670-9.

19. Marciscano, A.E., and Anandasabapathy, N. (2021). The role of dendritic cells in cancer and anti-tumor immunity. Semin. Immunol. *52*, 101481. https://doi.org/10.1016/j.smim.2021.101481.

20. Glaviano, A., Foo, A.S.C., Lam, H.Y., Yap, K.C.H., Jacot, W., Jones, R.H., Eng, H., Nair, M.G., Makvandi, P., Geoerger, B., et al. (2023). PI3K/AKT/mTOR signaling transduction pathway and targeted therapies in cancer. Mol. Cancer *22*, 138. https://doi.org/10.1186/s12943-023-01827-6.

21. Paplomata, E., and O'Regan, R. (2014). The PI3K/AKT/mTOR pathway in breast cancer: targets, trials and biomarkers. Ther. Adv. Med. Oncol. *6*, 154–166. https://doi.org/10.1177/1758834014530023.

22. Kim, I.A., Bae, S.S., Fernandes, A., Wu, J., Muschel, R.J., McKenna, W.G., Birnbaum, M.J., and Bernhard, E.J. (2005). Selective inhibition of Ras, phosphoinositide 3 kinase, and Akt isoforms increases the radiosensitivity of human carcinoma cell lines. Cancer Res. *65*, 7902–7910. https://doi.org/10.1158/0008-5472.Can-05-0513.

23. Marks, D.K., Gartrell, R.D., El Asmar, M., Boboila, S., Hart, T., Lu, Y., Pan, Q., Yu, J., Hibshoosh, H., Guo, H., et al. (2020). Akt Inhibition Is Associated With Favorable Immune Profile Changes Within the Tumor Microenvironment of Hormone Receptor Positive, HER2 Negative Breast Cancer. Front. Oncol. *10*, 968. https://doi.org/10.3389/fonc.2020.00968.

24. Kaneda, M.M., Messer, K.S., Ralainirina, N., Li, H., Leem, C.J., Gorjestani, S., Woo, G., Nguyen, A.V., Figueiredo, C.C., Foubert, P., et al. (2016). PI3Kγ is a molecular switch that controls immune suppression. Nature *539*, 437–442. https://doi.org/10.1038/nature19834.

25. Chang, W.I., Han, M.G., Kang, M.H., Park, J.M., Kim, E.E., Bae, J., Ahn, S., and Kim, I.A. (2021). PI3Kαδ Inhibitor Combined With Radiation Enhances the Antitumor Immune Effect of Anti-PD1 in a Syngeneic Murine Triple-Negative Breast Cancer Model. Int. J. Radiat. Oncol. Biol. Phys. *110*, 845–858. https://doi.org/10.1016/j.ijrobp.2021.01.025.

26. Han, M.G., Jang, B.S., Kang, M.H., Na, D., and Kim, I.A. (2021). PI3Kγδ inhibitor plus radiation enhances the antitumour immune effect of PD-1 blockade in syngenic murine breast cancer and humanised patient-derived xenograft model. Eur. J. Cancer *157*, 450–463. https://doi.org/10.1016/j.ejca.2021.08.029.

27. Covarrubias, A.J., Aksoylar, H.I., and Horng, T. (2015). Control of macrophage metabolism and activation by mTOR and Akt signaling. Semin. Immunol. *27*, 286–296. https://doi.org/10.1016/j.smim.2015.08.001.

28. Rückerl, D., Jenkins, S.J., Laqtom, N.N., Gallagher, I.J., Sutherland, T.E., Duncan, S., Buck, A.H., and Allen, J.E. (2012). Induction of IL-4Rα-dependent microRNAs identifies PI3K/Akt signaling as essential for IL-4-driven murine macrophage proliferation in vivo. Blood *120*, 2307–2316. https://doi.org/10.1182/blood-2012-02-408252.

29. Gris-Oliver, A., Palafox, M., Monserrat, L., Brasó-Maristany, F., Òdena, A., Sánchez-Guixé, M., Ibrahim, Y.H., Villacampa, G., Grueso, J., Parés, M., et al. (2020). Genetic Alterations in the PI3K/AKT Pathway and Baseline AKT Activity Define AKT Inhibitor Sensitivity in Breast Cancer Patient-derived Xenografts. Clin. Cancer Res. *26*, 3720–3731. https://doi.org/10.1158/1078-0432.Ccr-19-3324.

30. Liu, S., Wang, W., Hu, S., Jia, B., Tuo, B., Sun, H., Wang, Q., Liu, Y., and Sun, Z. (2023). Radiotherapy remodels the tumor microenvironment for enhancing immunotherapeutic sensitivity. Cell Death Dis. *14*, 679. https://doi.org/10.1038/s41419-023-06211-2.

31. Schmid, P., Adams, S., Rugo, H.S., Schneeweiss, A., Barrios, C.H., Iwata, H., Diéras, V., Hegg, R., Im, S.A., Shaw Wright, G., et al. (2018). Atezolizumab and Nab-Paclitaxel in Advanced Triple-Negative Breast Cancer. N. Engl. J. Med. *379*, 2108–2121. https://doi.org/10.1056/NEJMoa1809615.

32. Vito, A., Salem, O., El-Sayes, N., MacFawn, I.P., Portillo, A.L., Milne, K., Harrington, D., Ashkar, A.A., Wan, Y., Workenhe, S.T., et al. (2021). Immune checkpoint blockade in triple negative breast cancer influenced by B cells through myeloid-derived suppressor cells. Commun. Biol. *4*, 859. https://doi.org/10.1038/s42003-021-02375-9.

33. Hua, H., Zhang, H., Chen, J., Wang, J., Liu, J., and Jiang, Y. (2021). Targeting Akt in cancer for precision therapy. J. Hematol. Oncol. *14*, 128. https://doi.org/10.1186/s13045-021-01137-8.

34. Coleman, N., Moyers, J.T., Harbery, A., Vivanco, I., and Yap, T.A. (2021). Clinical Development of AKT Inhibitors and Associated Predictive Biomarkers to Guide Patient Treatment in Cancer Medicine. Pharmgenomics. Pers. Med. *14*, 1517–1535. https://doi.org/10.2147/pgpm.S305068.

35. Zhang, M., He, Y., Sun, X., Li, Q., Wang, W., Zhao, A., and Di, W. (2014). A high M1/M2 ratio of tumor-associated macrophages is associated with extended survival in ovarian cancer patients. J. Ovarian Res. *7*, 19. https://doi.org/10.1186/1757-2215-7-19.

36. Italiani, P., and Boraschi, D. (2014). From Monocytes to M1/M2 Macrophages: Phenotypical vs. Functional Differentiation. Front. Immunol. *5*, 514. https://doi.org/10.3389/fimmu.2014.00514.

37. Weisser, S.B., McLarren, K.W., Voglmaier, N., van Netten-Thomas, C.J., Antov, A., Flavell, R.A., and Sly, L.M. (2011). Alternative activation of macrophages by IL-4 requires SHIP degradation. Eur. J. Immunol. *41*, 1742–1753. https://doi.org/10.1002/eji.201041105.

38. Creighton, C.J. (2007). A gene transcription signature of the Akt/mTOR pathway in clinical breast tumors. Oncogene *26*, 4648–4655. https://doi.org/10.1038/sj.onc.1210245.

39. Martinez, F.O., Gordon, S., Locati, M., and Mantovani, A. (2006). Transcriptional profiling of the human monocyte-to-macrophage differentiation and polarization: new molecules and patterns of gene expression. J. Immunol. *177*, 7303–7311. https://doi.org/10.4049/jimmunol.177.10.7303.

OMTON, Volume *33*

Supplemental information

Akt inhibition enhances the antitumor efficacy of immune checkpoint blockades and radiotherapy in a syngeneic breast cancer model

Nawon Park, Seung Hyuck Jeon, Yoomin Kim, Seongmin Kim, and In Ah Kim

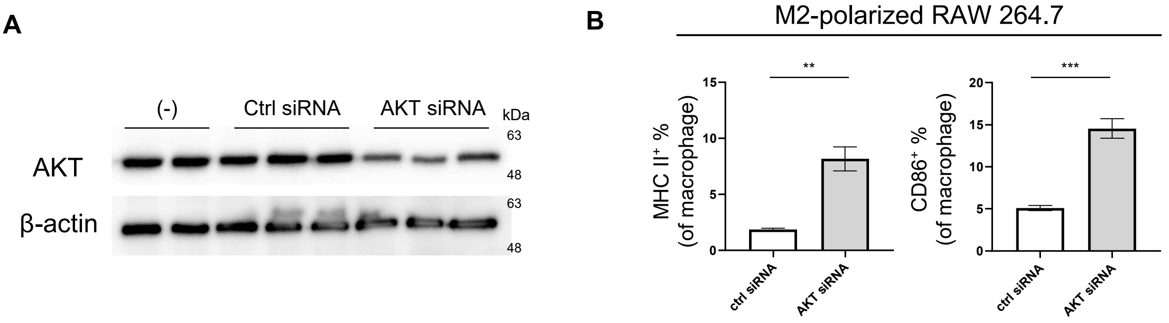

Figure S1. MHCII and CD86 expression following Akt siRNA treatment

RAW 264.7 cells were polarized into M2 macrophages and transfected with Ctrl siRNA or Akt siRNA. (A) Western blot analysis of AKT and β-actin expression. (B) Flow cytometry analysis of MHC II and CD86 expression after siRNA treatment. Positive gating for MHCII and CD86 was defined using M0 macrophages (negative control) with a 5% threshold. Data represent the mean ± S.E.M (n = 4). *P<0.05, **P<0.01, ***P<0.001, ****P<0.0001.

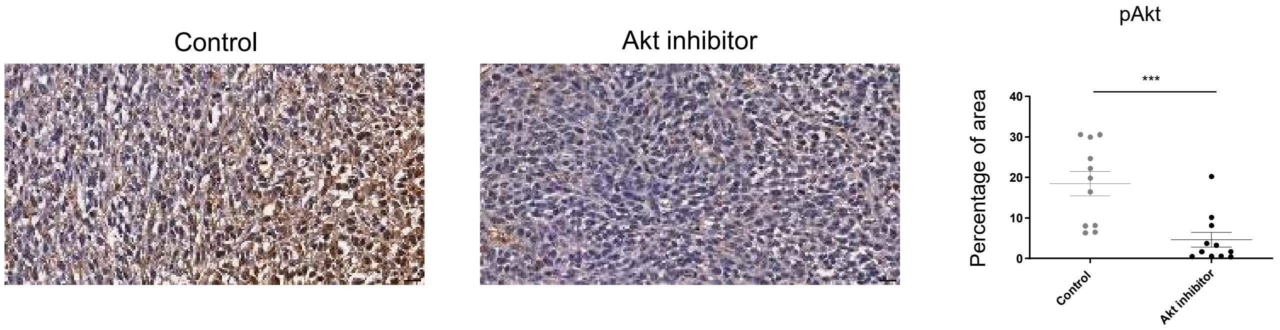

Figure S2. Phospho-Akt (Ser473) expression by IHC in primary tumors.
IHC staining for pAkt in primary tumor sections. Scale bars, 20 µm. Data represent the mean ± S.E.M (n = 11). *P<0.05, **P<0.01, ***P<0.001, ****P<0.0001.

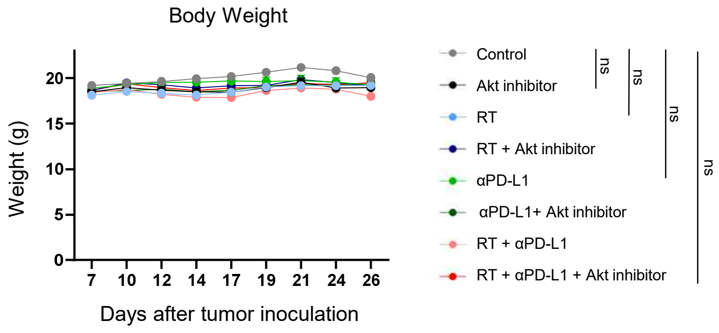

Figure S3. Body weight changes during treatment following RT, αPD-L1 and Akt inhibitor.
Body weight changes during treatment. Data represent the mean ± S.E.M (n = 5). *P<0.05, **P<0.01, ***P<0.001, ****P<0.0001 (two-way ANOVA with multiple comparisons at day 26).

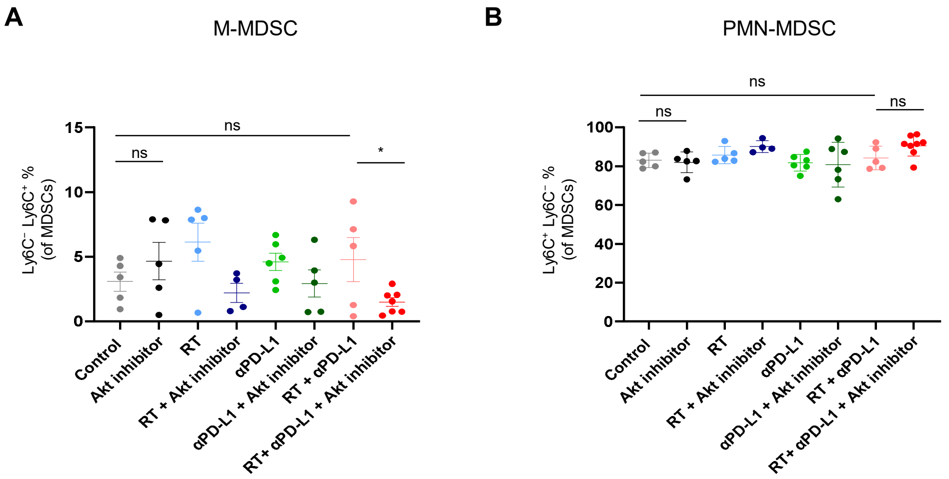

Figure S4. Phenotypic profiling of M-MDSCs and PMN-MDSCs in primary tumors following RT, αPD-L1 and Akt inhibitor.

Frequency of (A) M-MDSC ($CD45^+$, $CD11c^-$, $CD11b^+$, $F4/80^-$, $Ly6G^-$, $Ly6C^+$) and (B) PMN-MDSC ($CD45^+$, $CD11c^-$, $CD11b^+$, $F4/80^-$, $Ly6G^+$, $Ly6C^-$) in primary tumor. All data represent the mean ± S.E.M (n = 4~7). *P<0.05, **P<0.01, ***P<0.001, ****P<0.0001.

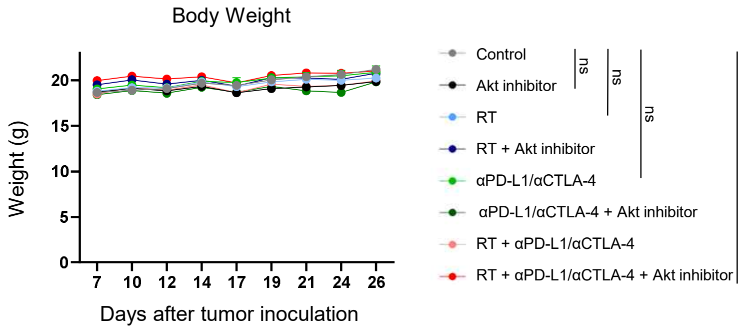

Figure S5. Body weight changes during treatment following RT, αPD-L1/αCTLA-4 and Akt inhibitor.
Body weight changes during treatment. All data represent the mean ± S.E.M (n = 5). *P<0.05, **P<0.01, ***P<0.001, ****P<0.0001 (two-way ANOVA with multiple comparisons at day 26).

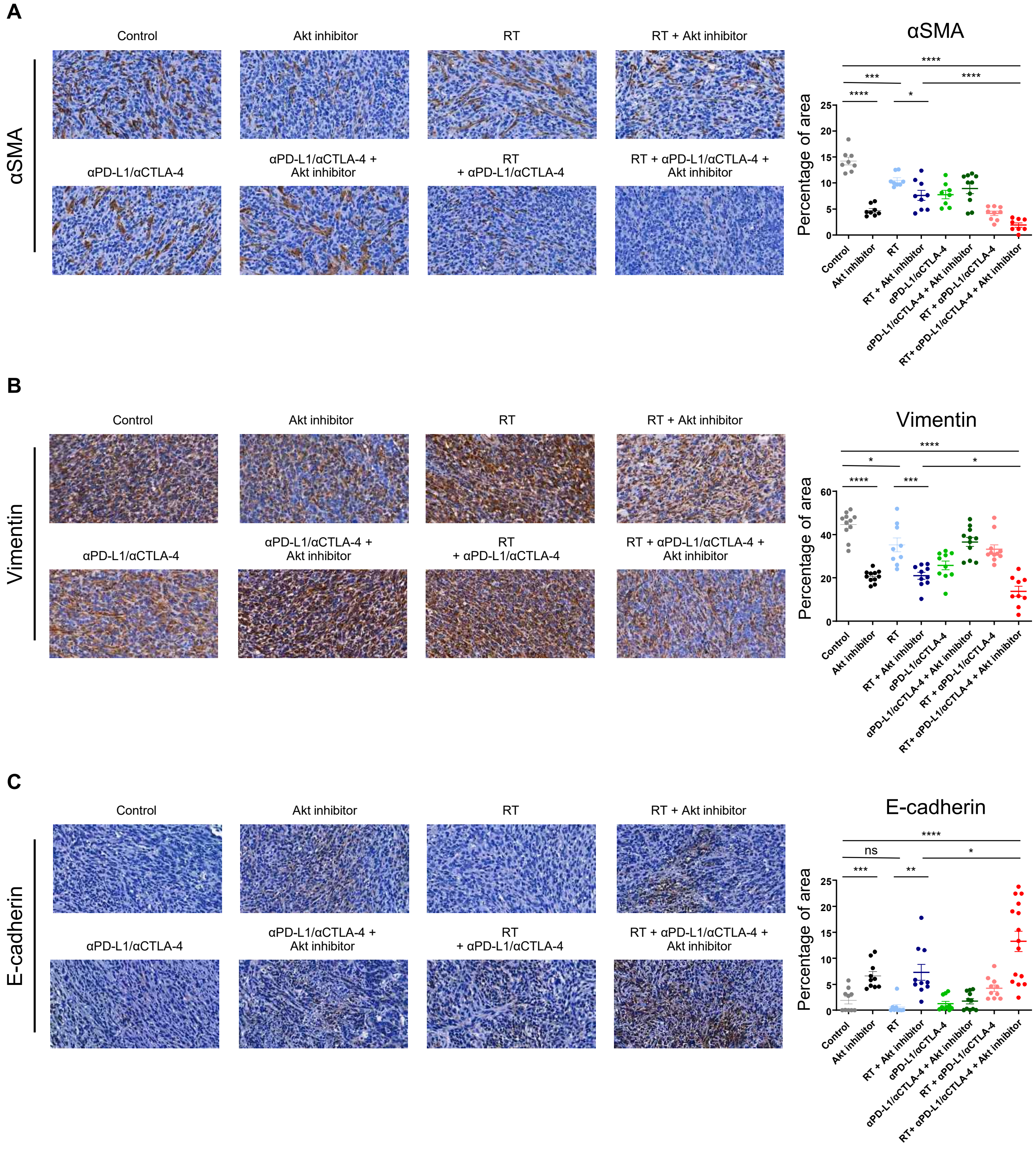

Figure S6. αSMA, vimentin and E-cadherin expression by IHC in primary tumors.
IHC staining of tumor sections for (A) αSMA, (B) vimentin, (C) E-cadherin. Scale bars, 20 μm. All data represent the mean ± S.E.M (n = 8~15). *P<0.05, **P<0.01, ***P<0.001, ****P<0.0001.

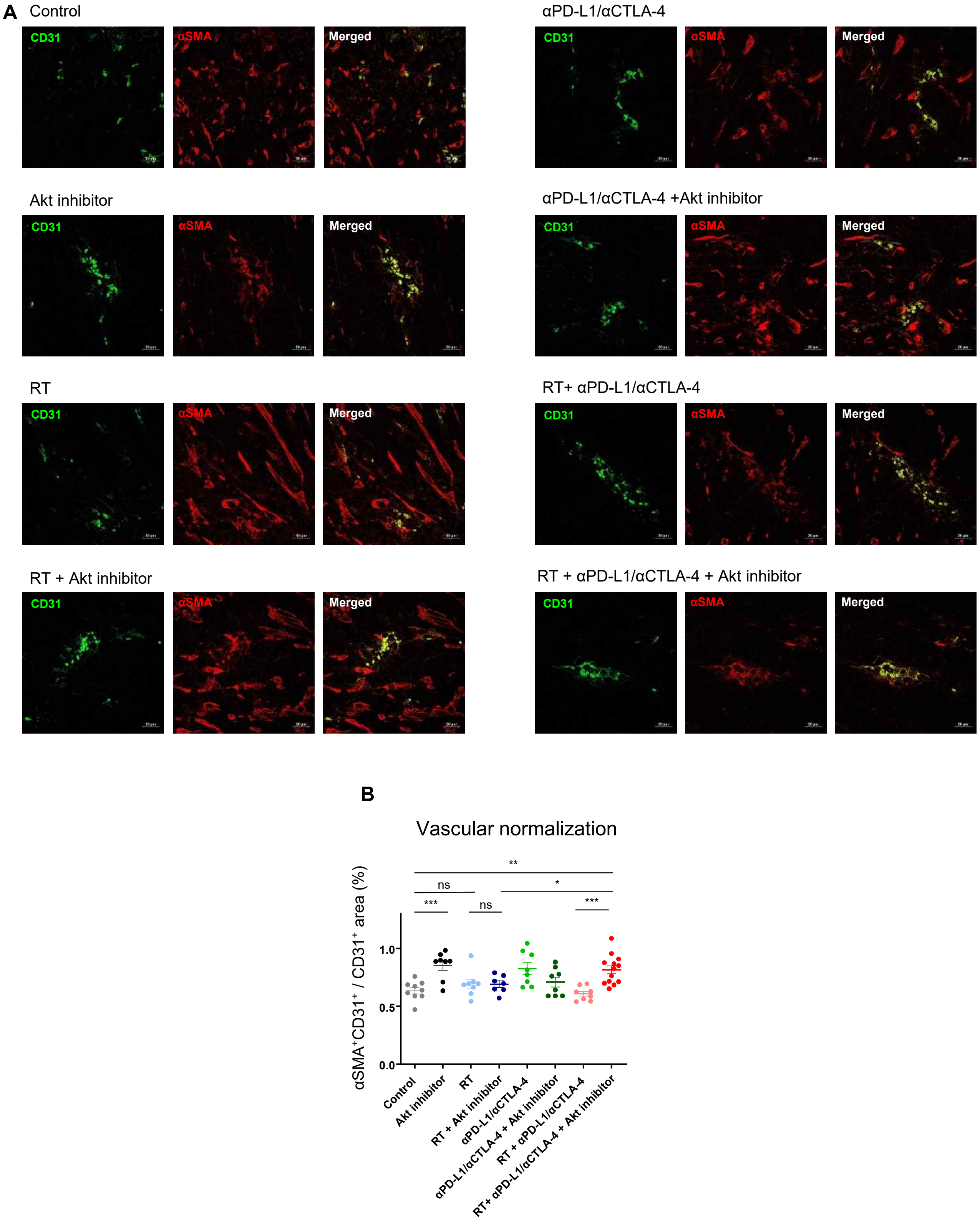

Figure S7. Colocalization of CD31 and αSMA in tumor vasculature.
(A) Immunofluorescence staining images of CD31 (green) and αSMA (red); yellow indicates colocalization. (B) Quantification of αSMA$^+$CD31$^+$/CD31$^+$ areas. Scale bars, 20 μm. All data represent the mean ± S.E.M (n = 7~13). *P*<0.05, **P*<0.01, ***P*<0.001, ****P*<0.0001.

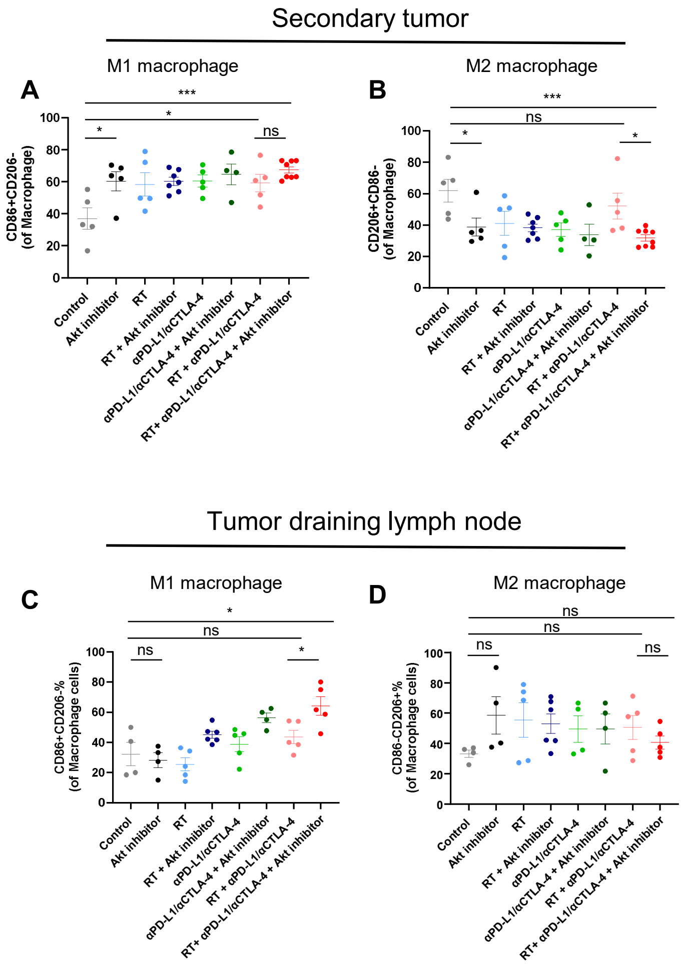

Figure S8. Macrophage profiling in the secondary tumor and tumor draining lymph node following RT, αPD-L1/αCTLA-4 and Akt inhibitor.

Frequency of (A) M1 and (B) M2 macrophages in the secondary tumor. Frequency of (C) M1, (D) M2 macrophages in the tumor-draining lymph node. All data represent the mean ± S.E.M (n = 4~6). *P<0.05, **P<0.01, ***P<0.001, ****P<0.0001.

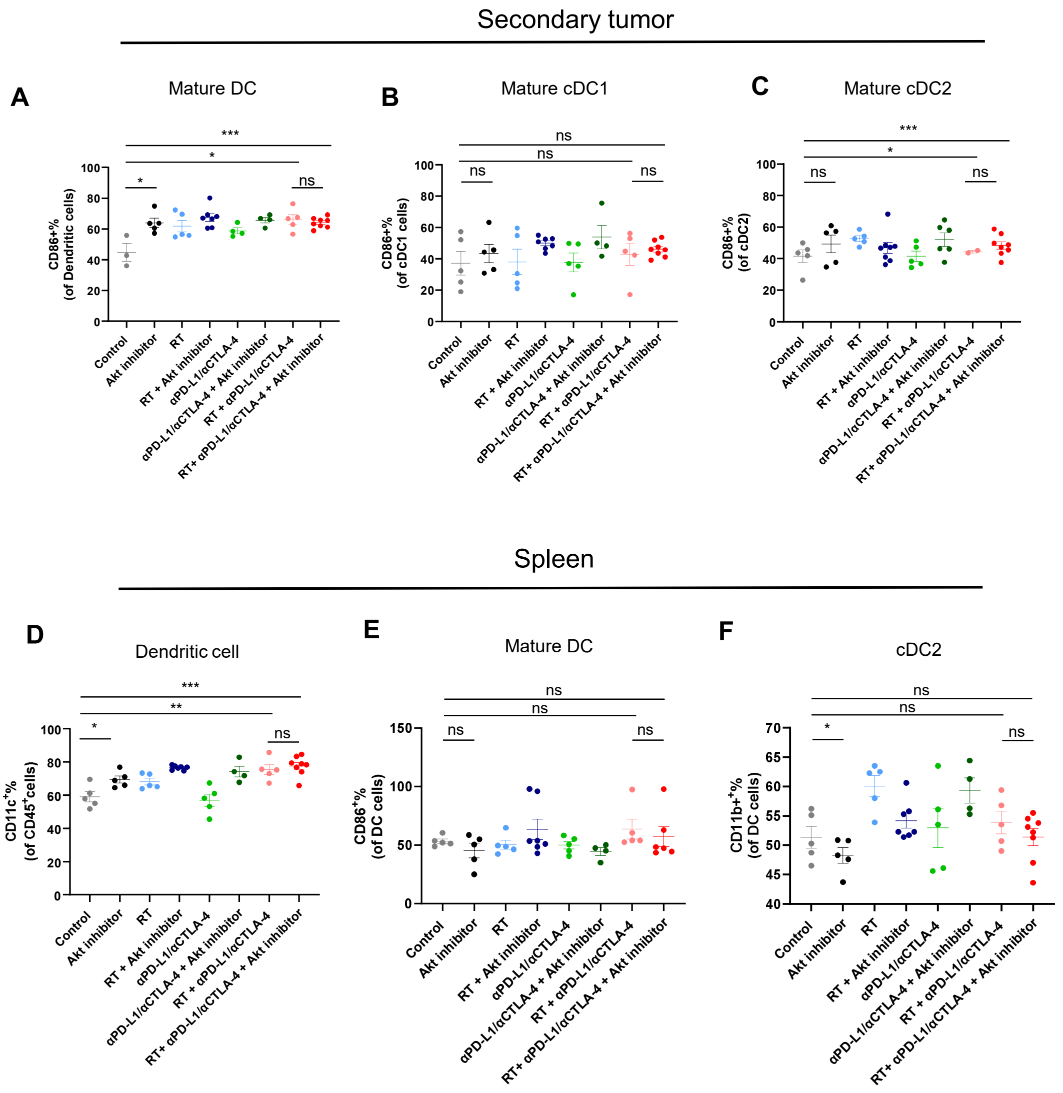

Figure S9. DCs profiling in the secondary tumor and spleen following RT, αPD-L1/αCTLA-4 and Akt inhibitor. Frequency of (A) CD86^{+} dendritic cells, (B) Frequency of CD86^{+} cDC1, (C) Frequency of CD86^{+} cDC2 in the secondary tumor. Frequency of (D) Dendritic cell (CD45^{+}, CD11c^{+}), (E) CD86^{+} DC, (F) cDC2 in the spleen. All data represent the mean ± S.E.M (n = 4~8). *P<0.05, **P<0.01, ***P<0.001, ****P<0.0001.

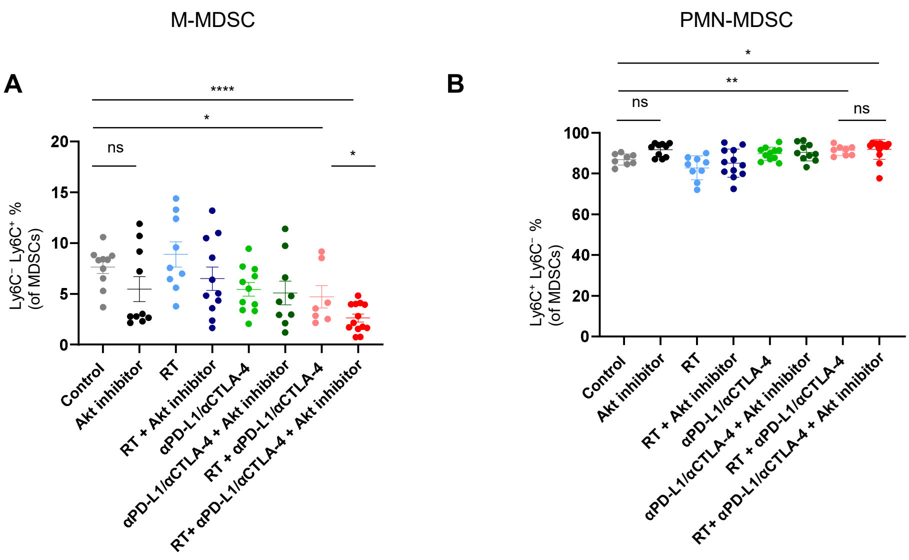

Figure S10. Phenotypic profiling of M-MDSCs and PMN-MDSCs in primary tumors following RT, αPD-L1/αCTLA-4 and Akt inhibitor.

Frequency of (A) M-MDSC and (B) PMN-MDSC in primary tumor. All data represent the mean ± S.E.M (n = 7~13). *P<0.05, **P<0.01, ***P<0.001, ****P<0.0001.
